# Lessons from assembling a microbial natural product and pre-fractionated extract library in an academic laboratory

**DOI:** 10.1093/jimb/kuad042

**Published:** 2023-12-05

**Authors:** Michael A Cook, Daniel Pallant, Linda Ejim, Arlene D Sutherland, Xiaodong Wang, Jarrod W Johnson, Susan McCusker, Xuefei Chen, Maya George, Sommer Chou, Kalinka Koteva, Wenliang Wang, Christian Hobson, Dirk Hackenberger, Nicholas Waglechner, Obi Ejim, Tracey Campbell, Ricardo Medina, Lesley T MacNeil, Gerard D Wright

**Affiliations:** Department of Biochemistry and Biomedical Sciences, M.G. DeGroote Institute for Infectious Disease Research, DeGroote School of Medicine, McMaster University, 1280 Main Street West, Hamilton, ON L8S 4K1, Canada; Department of Biochemistry and Biomedical Sciences, M.G. DeGroote Institute for Infectious Disease Research, DeGroote School of Medicine, McMaster University, 1280 Main Street West, Hamilton, ON L8S 4K1, Canada; Department of Biochemistry and Biomedical Sciences, M.G. DeGroote Institute for Infectious Disease Research, DeGroote School of Medicine, McMaster University, 1280 Main Street West, Hamilton, ON L8S 4K1, Canada; Department of Biochemistry and Biomedical Sciences, M.G. DeGroote Institute for Infectious Disease Research, DeGroote School of Medicine, McMaster University, 1280 Main Street West, Hamilton, ON L8S 4K1, Canada; Department of Biochemistry and Biomedical Sciences, M.G. DeGroote Institute for Infectious Disease Research, DeGroote School of Medicine, McMaster University, 1280 Main Street West, Hamilton, ON L8S 4K1, Canada; Department of Biochemistry and Biomedical Sciences, M.G. DeGroote Institute for Infectious Disease Research, DeGroote School of Medicine, McMaster University, 1280 Main Street West, Hamilton, ON L8S 4K1, Canada; Department of Biochemistry and Biomedical Sciences, M.G. DeGroote Institute for Infectious Disease Research, DeGroote School of Medicine, McMaster University, 1280 Main Street West, Hamilton, ON L8S 4K1, Canada; Department of Biochemistry and Biomedical Sciences, M.G. DeGroote Institute for Infectious Disease Research, DeGroote School of Medicine, McMaster University, 1280 Main Street West, Hamilton, ON L8S 4K1, Canada; Department of Biochemistry and Biomedical Sciences, M.G. DeGroote Institute for Infectious Disease Research, DeGroote School of Medicine, McMaster University, 1280 Main Street West, Hamilton, ON L8S 4K1, Canada; Department of Biochemistry and Biomedical Sciences, M.G. DeGroote Institute for Infectious Disease Research, DeGroote School of Medicine, McMaster University, 1280 Main Street West, Hamilton, ON L8S 4K1, Canada; Farncombe Family Digestive Health Research Institute, McMaster University, 1280 Main Street West, Hamilton, ON L8S 4L8, Canada; Department of Biochemistry and Biomedical Sciences, M.G. DeGroote Institute for Infectious Disease Research, DeGroote School of Medicine, McMaster University, 1280 Main Street West, Hamilton, ON L8S 4K1, Canada; Department of Biochemistry and Biomedical Sciences, M.G. DeGroote Institute for Infectious Disease Research, DeGroote School of Medicine, McMaster University, 1280 Main Street West, Hamilton, ON L8S 4K1, Canada; Department of Biochemistry and Biomedical Sciences, M.G. DeGroote Institute for Infectious Disease Research, DeGroote School of Medicine, McMaster University, 1280 Main Street West, Hamilton, ON L8S 4K1, Canada; Department of Biochemistry and Biomedical Sciences, M.G. DeGroote Institute for Infectious Disease Research, DeGroote School of Medicine, McMaster University, 1280 Main Street West, Hamilton, ON L8S 4K1, Canada; Department of Biochemistry and Biomedical Sciences, M.G. DeGroote Institute for Infectious Disease Research, DeGroote School of Medicine, McMaster University, 1280 Main Street West, Hamilton, ON L8S 4K1, Canada; College of Medicine, Enugu State University of Science and Technology, Agbani, Enugu State, PMB 01660, Nigeria; Department of Biochemistry and Biomedical Sciences, M.G. DeGroote Institute for Infectious Disease Research, DeGroote School of Medicine, McMaster University, 1280 Main Street West, Hamilton, ON L8S 4K1, Canada; Department of Microbiology, Chemical Bioactive Center, Central University Marta Abreu de las Villas, Santa Clara 54830, Villa Clara, Cuba; Department of Biochemistry and Biomedical Sciences, M.G. DeGroote Institute for Infectious Disease Research, DeGroote School of Medicine, McMaster University, 1280 Main Street West, Hamilton, ON L8S 4K1, Canada; Farncombe Family Digestive Health Research Institute, McMaster University, 1280 Main Street West, Hamilton, ON L8S 4L8, Canada; Department of Biochemistry and Biomedical Sciences, M.G. DeGroote Institute for Infectious Disease Research, DeGroote School of Medicine, McMaster University, 1280 Main Street West, Hamilton, ON L8S 4K1, Canada

**Keywords:** Natural product extract library, Antimicrobial screening, Actinomycete collection, Dereplication, Fractionation

## Abstract

Microbial natural products are specialized metabolites that are sources of many bioactive compounds including antibiotics, antifungals, antiparasitics, anticancer agents, and probes of biology. The assembly of libraries of producers of natural products has traditionally been the province of the pharmaceutical industry. This sector has gathered significant historical collections of bacteria and fungi to identify new drug leads with outstanding outcomes—upwards of 60% of drug scaffolds originate from such libraries. Despite this success, the repeated rediscovery of known compounds and the resultant diminishing chemical novelty contributed to a pivot from this source of bioactive compounds toward more tractable synthetic compounds in the drug industry. The advent of advanced mass spectrometry tools, along with rapid whole genome sequencing and *in silico* identification of biosynthetic gene clusters that encode the machinery necessary for the synthesis of specialized metabolites, offers the opportunity to revisit microbial natural product libraries with renewed vigor. Assembling a suitable library of microbes and extracts for screening requires the investment of resources and the development of methods that have customarily been the proprietary purview of large pharmaceutical companies. Here, we report a perspective on our efforts to assemble a library of natural product-producing microbes and the establishment of methods to extract and fractionate bioactive compounds using resources available to most academic labs. We validate the library and approach through a series of screens for antimicrobial and cytotoxic agents. This work serves as a blueprint for establishing libraries of microbial natural product producers and bioactive extract fractions suitable for screens of bioactive compounds.

**One-Sentence Summary:**

Natural products are key to discovery of novel antimicrobial agents: Here, we describe our experience and lessons learned in constructing a microbial natural product and pre-fractionated extract library.

## Graphical Abstract

**Graphical Abstract:**
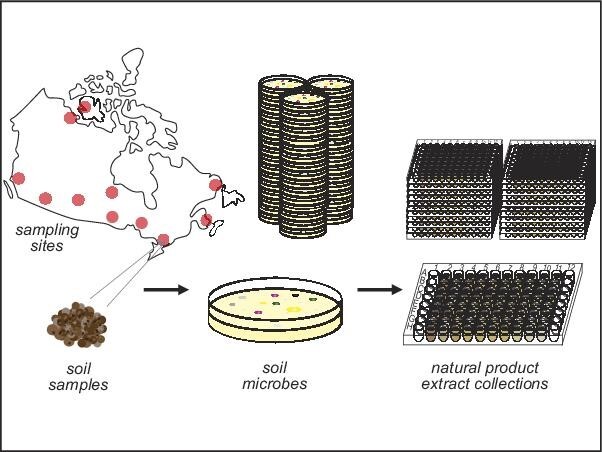

## Introduction

Natural products are specialized metabolites produced by living organisms. They are sources of antimicrobial agents, insecticides, herbicides, various drugs, and vital molecular probes of biological processes (Katz & Baltz, 2016; Walsh & Tang, 2017). Bioactive natural products have historically been discovered based on traditional medical practices, for example salicylic acid from willow bark as an analgesic, or as the result of serendipitous discoveries such as penicillin from environmental molds. The discovery of antibiotics such as gramicidin and streptomycin from soil bacteria in the early 1940s ushered in an era of systematic collecting of environmental microbes followed by screens of extracts for bioactivity by the pharmaceutical and crop protection industries, a strategy termed the Waksman Platform (Lewis, 2013).

The Waksman platform was groundbreaking for drug discovery. This strategy identified most of the antibiotic scaffolds in current use, many antifungal and antiparasitic agents, anticancer compounds, cholesterol-lowering agents, immunosuppressants, and more (Lewis, 2020). Microbes were also productive sources of herbicides (e.g. bialaphos) and insecticides (e.g. spinosyns) (Demain & Sanchez, 2009). As a result of these successes, most large pharmaceutical and crop protection companies accumulated in-house libraries of thousands of environmental bacteria and fungi. For decades, organic and aqueous extracts of these isolates were employed in activity-based screens, resulting in many high-value products.

Despite this success, microbial natural products have drawbacks (Butler, 2004; Atanasov et al., 2021). These compounds are generated in complex mixtures of metabolites and must be purified by bespoke methods, the quantity of active compound is often low, requiring significant strain optimization to improve yield, and similar chemical scaffolds are frequently re-isolated (the dereplication problem). By the end of the 20^th^ century, due to these challenges along with the advent of competing sources of compounds such as combinatorial platforms to generate large libraries of molecules, interest in natural products began to wane.

In parallel with the pivot away from microbial natural products by industry, several innovations began to fuel renewed interest in this source of bioactive chemical matter. Genome sequencing of natural product-producing bacteria revealed that most bacteria and fungi have dozens of genetic programs for producing natural products and that many are underexpressed under lab conditions, meaning that there is significant untapped bioactive potential in libraries of microbes (Pepler et al., 2022). Furthermore, emerging tools such as Clustered Regularly Interspaced Short Palindromic Repeats (CRISPR)-mediated inactivation of the biosynthesis of commonly expressed compounds offers new strategies to re-mine microbes for rare metabolites (Culp et al., 2019). The development of rapid mass spectrometry tools and community-based platforms for data analysis, such as Global Natural Products Social (GNPS) molecular networking (Nothias et al., 2020), as well as cell-based arrays of antibiotic resistance elements (Cox et al., 2017), are helping to address the dereplication problem. Synthetic biology methods offer modern approaches to tackle strain improvement and minimization of unwanted side products (Xu et al., 2020; Alam et al., 2021). These advances provide confidence that a renaissance in microbial natural product drug discovery is underway.

While these advances offer new routes to explore natural products, the large collections assembled by industry over decades often remain proprietary and inaccessible to academic labs. A recent welcome exception is the donation of Pfizer's historic collection to the Natural Product Library Initiative at the Scripps Research Institute (Steele et al., 2019). Over 20 years ago, our team began assembling the Wright Actinomycete Collection (WAC), initially focusing on isolating highly gifted natural product-producing actinomycetes from soil. The collection has grown to include ∼12 000 isolates of actinomycetes and other bacteria and filamentous fungi isolated from diverse environments, resulting in the isolation and characterization of several new bioactive compounds. Here, we report our strategy and insights gained from establishing a collection of natural product producers in an academic setting and the application of a cost-effective method of partial fractionation of extracts to improve the output of high-throughput screens.

## Materials and Methods

### Isolation of Environmental Bacteria and Fungi

The original isolates in the WAC were prepared following the protocols detailed by D'Costa et al. (D'Costa et al., 2006). Our methodology has evolved with experience, and various media and antibiotic selection conditions are employed to isolate additional diverse bacterial and fungal phyla. A consensus method is presented below. Further details on a range of pre-treatment conditions, isolation media, and specialized procedures for isolating fungal endophytes or insect microbiota isolates are described in Supplementary Tables S1–S3 and the frequency of application of each pre-treatment or isolation media is indicated in Supplementary Fig. S1A and S1B.

Soil samples were dried in sterile petri dishes at room temperature for 5–7 days. For each sample, 1 g of soil was resuspended in 10 mL of sterile distilled deionized water (ddH_2_O) and mixed by vortexing. Three 10-fold serial dilutions with sterile ddH_2_O were prepared, and 100 µL of each suspension was plated on isolation media. For a large proportion of the collection (Supplementary Fig. S1B), strains were isolated on Streptomyces Isolation Media (SIM) consisting of 0.2 g casein, 0.5 g starch, 0.25 g KNO_3_, 0.1 g K_2_HPO_4_, 0.05 g MgSO_4_, 0.05 g CaCO_3_, 7.5 g agar in 500 mL ddH_2_O, supplemented with cycloheximide (20 µg/mL) to inhibit fungal growth. Plates were incubated at room temperature without light to mimic environmental conditions. Putative actinomycetes were identified as sporulating colonies, commonly producing a distinctive soil-like odor, and purified to apparent homogeneity by serial passage on SIM. Strains were subsequently analyzed by Gram stain and Gram-positive, filamentous isolates, with a synchronous segmentation pattern characteristic of actinomycetes (Kieser et al., 2000) were preserved.

Spore stocks were prepared in duplicate, using a modified protocol (Kieser et al., 2000). Briefly, a single colony was inoculated into a 5 mL culture of Soygrit Vegetative Media (SVM, 7.5 g glucose, 10 g potato starch, 7.5 g soybean grits, 5 g yeast extract, 1 g CaCO_3_, 5 g corn steep liquor, pH 6.5, in 500 mL ddH_2_O) (Marshall & Wright, 1996) containing a metal spring to promote aeration, and incubated at 30°C for 2–4 days. A confluent lawn was prepared on Bennett's Agar (5 g potato starch, 1 g casamino acids [Biobasic], 0.9 g yeast extract [Thermo Fisher Scientific], 7.5 g agar in 500 mL ddH_2_O, pH to 6.8; add 1 mL Czapek Mineral Mix [10 g KCl, 10 g MgSO_4_•7H_2_O, 12 g NaNO_3_, 0.2 g FeSO4, 200 µL concentrated HCl in 100 mL] after autoclaving) using 700 µL of each culture. Plates were incubated at 30°C until dense sporulation was evident (approximately 7–10 days). A layer of spores was removed from the agar surface, resuspended in 9 mL of sterile ddH_2_O by vortexing, and gravity filtered through sterile filters of cotton wool (Kieser et al., 2000). The filtrate was centrifuged at 3000 rpm for 15 min, and the subsequent pellet was resuspended in 1 mL of sterile 20% glycerol for storage at −80°C.

Fungal isolates were cultured in potato dextrose (PD) media (Bioshop). Non-spore-forming microorganisms were cultured in liquid Bennett's media. Strains were stored as 20% glycerol stocks at −80°C.

### Isolate Characterization

Oligonucleotide primers are described in Supplementary Table S4. Microbial DNA samples for Polymerase Chain Reaction (PCR) were prepared from a single colony with PrepMan Ultra (Thermo Fisher Scientific) according to the manufacturer's instructions. BOX-PCR was performed as described using primer BOXA1R (Lanoot et al., 2004). 16S PCR was performed with primers 16_BAC_F and 16_BAC_R as described (Heuer et al., 1997). 18S PCR was performed as described with primers 18S_For and 18S_Rev (Borneman & Hartin, 2000). Sanger sequencing was performed at McMaster Genomics Facility.

Fungal microscopy was performed on an Evos XL Core microscope with a 100X objective lens on cells stained with lactophenol cotton blue stain (Thermo Fisher Scientific).

### Genome Analyses

The WAC phylogenetic tree was generated using the Genome Taxonomy Database Toolkit (GTDB-Tk v2.2.6 de_novo_wf, (Chaumeil et al., 2022)) with all unique sequenced WAC genomes as input alongside the GTDB p__Actinobacteria reference genomes and GCF_003569045 (*Aggregatilinea lenta*) as an outgroup. The tree was visualized using the Interactive Tree of Life (iTOL v6.7.6, (Letunic & Bork, 2021)), and clades were roughly colored according to taxonomic orders with > 100 leaves. Biosynthetic gene clusters were identified and visualized using antiSMASH version 7 (Blin et al., 2023).

### Culture of Isolates and Generation of the Natural Product Library (NPL)

WAC isolates were applied to Bennett's agar using an autoclaved sterile wooden applicator and incubated for 6 days at 30°C to assess for culture purity. Strains were inoculated in 3 mL of Streptomyces Antibiotic Activity Media (SAM) (15 g glucose, 15 g Phytone [soya peptone;Thermo Fisher Scientific], 5 g NaCl, 1 g yeast extract [Thermo Fisher Scientific], 1 g CaCO_3_, 2.5 mL glycerol, ddH_2_O to 1 L, pH to 6.8) containing a single 3 mm glass bead and incubated for 6 days at 30°C, shaking at 250 rpm.

SAM cultures were inoculated to fresh Bennett's agar in duplicate and incubated at 30°C for 6 days to generate a lawn of growth. These cultures were then pressed through an empty 50 mL syringe using a mechanical “can crusher” (MasterCrush, Carver's Olde Iron) and extracted with 10 mL methanol overnight, shaking at 250 rpm. The methanol extract was then passed through a milk filter (KenAG, model number D110) to remove solids and collected in 16 × 100 mm disposable borosilicate glass tubes. The extracts were evaporated overnight in a Genevac centrifugal evaporator (SP Scientific) and resuspended in dimethyl sulfoxide (DMSO). The crude extracts were combined and transferred to 2 mL centrifuge tubes. For fungal cultures, the same protocol was employed, but cells were cultured in potato dextrose media (Bioshop, with 20 g agar added for solid media).

Aliquots of the DMSO extracts were arrayed in 96-well daughter microtiter plates and stored at −30°C.

### Prefractionation Library (PFL)

NPL extracts were subjected to reverse-phased C18 chromatography using a medium-pressure Teledyne NextGen CombiFlash system to generate a library of partially purified fractions. The crude extract was applied to a sample load cartridge (#693873235, Teledyne) containing 1 g of packed C18 resin and separated on a 15.5 g HP C18 Gold column (RediSep Rf Gold C18 High-Performance Columns #692203334, Teledyne) equilibrated with 90% solution A (ddH_2_O and 0.1% formic acid): 10% solution B (acetonitrile + 0.1% formic acid). A volume of 13.5 mL (∼1 column volume, CV) fractions were collected across a linear gradient of solution B from 10% to 100% (Program: 3 CV, 10% B; 11 CV, 10–100% B; 5 CV, 100%−10% B; 2 CV, 10% B). Chromatography was performed at room temperature at a nominal flow rate of 30 mL/min, adapting for pressure. After chromatographic separation, the first 22 fractions were evaporated overnight by centrifugal evaporation (Genevac) with standard low-temperature (27°C) High Performance Liquid Chromatography (HPLC) fraction protocols.

Combiflash fractions were dissolved in DMSO to a total of 750 μL, combined as follows: Fraction 1 (3 CV, ∼40 mL); Fractions 2–7 (2 CV, ∼27 mL); Fraction 8 (7 CV, ∼95 mL).The fractions were added along with the crude extract and conditioned media (CM) to 96-well Costar polystyrene daughter microtiter plates and stored at −30°C.

### Generation of Conditioned Media Samples

Liquid Bennett's media (5 mL in a 50 mL centrifuge tube) was inoculated with a single colony. Three sterile 3 mm glass beads were added to prevent clumping. Cultures were shaken at 250 rpm and 30°C for 6 days with loose caps. After incubation, the conditioned medium was filtered through 0.22 µm polyethersulfone filters (Millex-GP) and added to the PFL plates.

### Cell-based High-Throughput Screens of Bacterial and Fungal Strains

High-throughput (HT) screens were performed using the PFL against the ESKAPE pathogens—*Enterobacter aerogenes* ATCC 13048, *Staphylococcus aureus* ATCC 29213, *Klebsiella pneumoniae* ATCC 33495, *Acinetobacter baumannii* ATCC 17978, *Pseudomonas aeruginosa* PAO1, *Enterococcus faecium* ATCC 19434; as well as *Escherichia coli* ATCC 25922; *E. coli* BW25113 *ΔbamBΔtolC; E. coli* BW25113 *ΔbamBΔtolC* pLacI-NDM-1; and the pathogenic yeast species *Candida albicans* ATCC 90028 and *Candida auris* CBS 10913.

Bacteria and yeast were assayed in cation-adjusted Mueller–Hinton Broth media (caMHB, Fisher Scientific) and RPMI-1640 media (R6504, Sigma–Aldrich), respectively. Before screening, bacterial and yeast cultures were grown 24 h at 37°C on caMHB and 48 h at 30°C on YPD, respectively. From these cultures, a final concentration of 0.0005 OD_625_ of bacteria or 0.000055 OD_530_ of yeast was obtained and used in screening.

HT screens were performed in 384-well microtiter plates (Corning 3701) with 49 μL of inoculated media and 1 μL of crude extracts/CM/fractions. Fractions, extracts, and inoculated media were dispensed to plates with a Biomek FX^P^ Integrated Liquid Handler. Plates were incubated for 24 h at 37°C and 48 h at 30°C for bacteria and yeast, respectively. Cell growth was quantified at OD_600_ on an EnVision (Molecular Devices), SpectraMax (Molecular Devices), or Biotek Neo microtiter plate reader. The raw data were analyzed and normalized in R.

### Cytotoxicity Screen

On day 1, HEK293 cells (ATCC CRL-1573) were seeded at 15 000 cells/well in 96-well tissue culture treated white plates (Corning 3917) in 100 µL Dulbecco Modified Eagle Medium (DMEM) supplemented with 10% fetal bovine serum, 2 mM l-glutamine, 100 units/mL penicillin, and 100 µg/mL streptomycin using the Formulatrix Tempest. Plates were incubated for 18 h at 37˚C under 5% CO2. Diluted extracts were prepared immediately before their addition: 99 μL of DMEM was dispensed into a sterile 96- well plate (Corning 3357) using the Formulatrix Tempest and 1 μL of compound from the NPL was added to each well using a Biomek FX^P^, for a final DMSO concentration of 1% A BioTek ELx405 was used to remove existing media from cells and the Biomek FX^P^ was used to add the previously prepared diluted extracts in DMEM to each well. Cells were incubated for an additional 48 h, after which cell viability was assessed using Promega Cell Titer Glo 2.0 reagent (Thermo Fisher Scientific). Cell Titer Glo (25 µL) was added directly to the media, the plates were shaken for 2 min and then incubated for 10 min at room temperature. The luminescence was read on a Neo2 plate reader (Biotek) using luminescence fiber (20 ms integration time). Controls were untreated cells and cells treated with DMSO only.

### *Caenorhabditis elegans* Activity Testing

Wild-type Bristol (N2) *C. elegans* eggs were collected by hypochlorite treatment (Stiernagle, 2006) and were age-synchronized to the first larval stage (L1) in S-basal medium (5.85 g NaCl, 1 g K_2_HPO_4_, 6 g KH_2_PO_4_, ddH_2_O to 1 L). Cholesterol was added to a final concentration of 5 mg/L following sterilization of the medium. Worms were sorted using a Union Biometrica COPAS Flow Pilot sorter to dispense 60 L1-stage larvae (in ∼60 μL of S-basal) into each well of a 96-well flat-bottom plate (Corning). Next, 89 μL of concentrated *E. coli* OP50 (equivalent to OD_600_ 4.5) in S-basal and 1 μL of PFL/NPL extract/fraction or DMSO were added using automated liquid handlers (Formulatrix Tempest and SPT LabTech Mosquito, respectively) for a total well volume of 150 μL. Plates were sealed with a gas-permeable film (VWR, 60941–086) and incubated at 20°C, 150 rpm for 6 days, after which time each well was imaged using a Nikon AZ100M microscope.

### Data Analysis

Data analysis was performed in R using custom scripts.

Raw data from bacterial and yeast screens, in 384-well format, were normalized by row and column and converted to *Z*-scores. Composite *Z*-scores, the average of replicate screen data, are presented. After the exclusion of plates with poor reproducibility, raw data from HEK cell screens in 96-well format were normalized by a modified row–row normalization approach (Supplementary Fig. S2) using trends across rows to correct for edge effects across columns. An additional corner-specific normalization step was included to mitigate spatial effects. Conditioned media data for all screens were excluded from subsequent analyses.

Subsequent analyses were performed with composite *Z*-score data. “Hits” were defined as a composite *Z*-score ≤2. Hierarchical clustering for Fig. 3 was performed on the complete set of composite Z score data per each WAC strain (including crude and all fraction data) to match closely related patterns of activity. Subsequently, WAC strains were roughly grouped by which fraction contained the maximal activity; as a result, the hierarchical structure is only approximate.

### Liquid Chromatography (LC)-Mass Spectrometry (MS)

High-resolution mass spectrometry of WAC extracts and fractions was recorded on an Agilent 6550 iFunnel Q-TOF mass spectrometer equipped with an inline Agilent 1290 HPLC system using electrospray ionization in positive or negative mode. Tandem MS/MS fragmentation of ions was performed on the same Q-TOF system with the indicated collision-induced dissociation energies. Chromatographic separation was performed on a ZORBAX Eclipse XD8-C8 column with solvent A (0.1% formic acid in water) and solvent B (acetonitrile with 0.1% formic acid). Samples were analyzed for UV absorbance as indicated.

Feature-based molecular networks were analyzed using the Global Natural Products Social Molecular Networking platform (GNPS, (Nothias et al., 2020)).

## Results

### Generation of the WAC Library

**Fig. 1.**
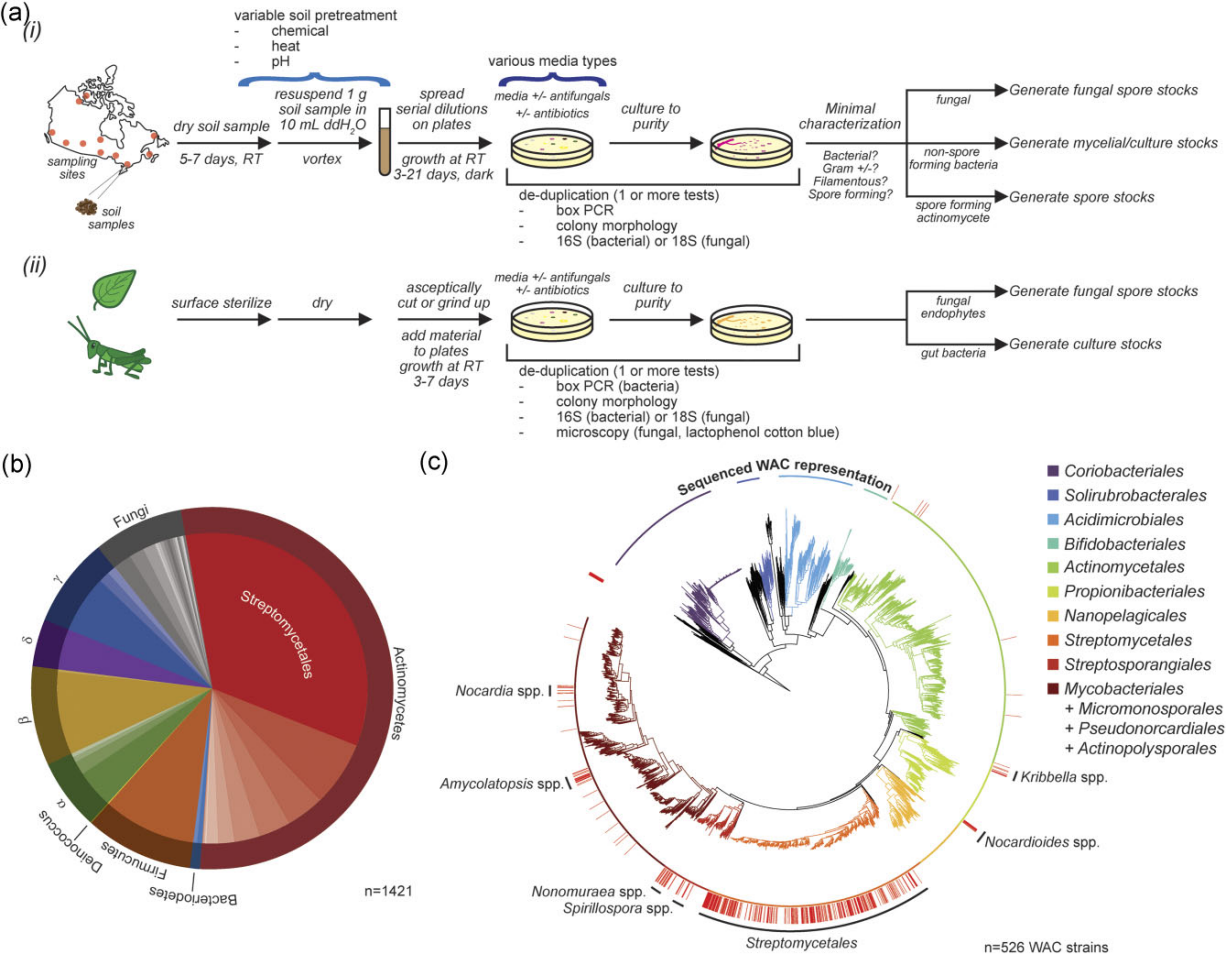
Construction and composition of the Wright Actinomycetes Collection (WAC). (a) Workflow for the isolation of bacterial and fungal strains from (i) soil and (ii) plant or insect samples. (i) Soil samples were collected from diverse environments across Canada and other countries not represented here. A range of pre-treatment conditions and culture conditions were employed to enrich for particular bacterial genera. (ii) Plant and insect samples were collected, surface sterilized, and prepared for isolation of fungal endophytes and bacteria from the insect microbiome, respectively. Pre-treatment conditions for soil, plant, and insect samples are described in more detail in the Supplementary Tables S1 and S2. Media conditions employed in isolation of microorganisms are described in Supplementary Table S3. (b) Overview of the composition of the WAC library, as determined by 16S bacterial and 18S fungal sequencing of a subset of the library (*n* = 1421 of 12 000 strains). (c) Composition of the WAC library, specifically within the phylum Actinobacteriota, as determined by whole genome sequencing. Strains were predominantly chosen for sequencing as biased by antimicrobial activity across greater than a decade of whole cell extract screening. Strains are roughly binned by predominant taxonomic orders within the tree. The red lines surrounding the tree indicate genome sequence of strains within the WAC library. Regions of the tree enriched for WAC strains are indicated.

The WAC collection of soil microbes was constructed over several years from environmental samples collected principally from diverse locations across Canada, the US, and through productive collaborations with colleagues in other countries, consistent with the Nagoya protocol on access and benefit sharing (https://www.cbd.int/abs/). Procedures for the isolation of microorganisms have varied and evolved to enrich for different bacterial or fungal genera. Our consensus methods for isolating organisms from soil, plant, or insect samples are presented in Fig. 1a. We employed pre-treatment conditions to select heat and chemical-resistant spores from actinomycetes and added pre-treatments and modulated media isolation conditions to expand diversity in bacterial and fungal species, endophytes, or insect microbiomes (Supplementary Tables S1–S3; Supplementary Fig. S1A and S1B).

A significant issue in building a collection of environmental microbes is avoiding strain duplication. While difficult to avoid entirely, particularly when isolating strains in independent batches, we have had reasonable success *within* batches with BOX-PCR (Supplementary Fig. S1C (Lanoot et al., 2004)). BOX-PCR employs primer binding at multiple common loci in bacterial genomes to generate a characteristic banding pattern. Strains with identical banding patterns and common colony morphology are likely the same species, and only one isolate is retained. In the case of fungal strains, a combination of colony morphology and microscopy (Supplementary Fig. S1D) is helpful to de-duplicate strains.

For a portion of the WAC isolates (∼12 %), we have sequenced the variable region of 16S (bacteria) or internally transcribed region of 18S rRNA (fungi). These data estimate the WAC library's composition (Fig. 1b). As expected, given the emphasis on spore-forming actinomycetes, these organisms dominate the collection, particularly from the order Streptomycetales. However, the collection also includes many Firmicutes; *α, β, γ*, and *δ*-proteobacteria, and filamentous fungi. Additionally, we have genome sequences for a subset of the WAC library (∼4%), chosen primarily for their antimicrobial activity (Fig. 1c). When included within a phylogenetic tree of phylum Actinobacteriota, we can see broad coverage of the order Streptomycetales, and lesser coverage of *Nocardia, Amycolatopsis, Nonomuraea, Spirillospora, Nocardioides*, and *Kribbella* genera. There are also areas of the tree with minimal coverage, representing avenues for future targeted collecting and exploration.

### Generation of the Natural Product Extract Library (NPL)

The WAC is an excellent source of bioactive compounds and has been employed by our team for a range of applications, including the identification of new anticancer stem cell agents and antimalarials (Benoit et al., 2021; Alder et al., 2022); co-culture identification of novel antifungal agents (Robbins et al., 2016); genome mining-based natural product discovery (Culp et al., 2020, 2022; Xu et al., 2020, 2022; Yarlagadda, Medina, Johnson, et al., 2020); and the exploration of environmental antimicrobial resistance (D'Costa et al., 2006). However, the principal use of the WAC has been in screens for novel antimicrobial agents and antibiotic adjuvants.

**Fig. 2.**
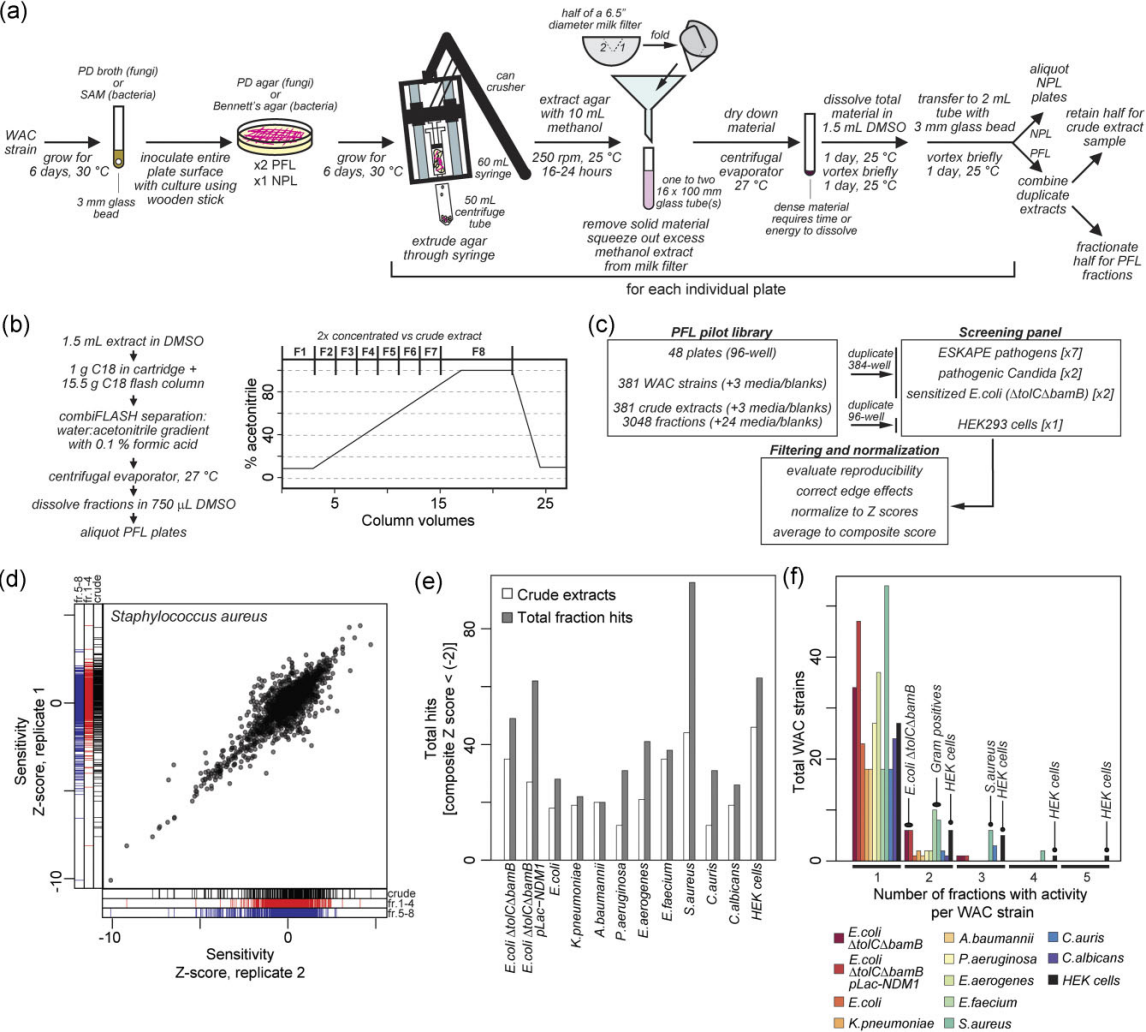
Generation of a natural product extract library (NPL) and prefractionation extract library (PFL) from the WAC and pilot screening against a panel of bacterial and fungal pathogens. (a) Workflow for generation of natural product extracts from bacterial and fungal strains. Depending on the application, either one (NPL) or two (PFL) plates are inoculated for extract preparation. To increase surface area for natural product extraction with methanol, agar cultures are extruded through syringes with the aid of a large can crusher. After extraction, half of a milk filter (to minimize absorption of solvent to the filter) is used to remove solid material from methanol extracts. The half filter is folded as indicated. When generating samples for the PFL, half of the final DMSO dissolved extracts are reserved as a “crude” sample, equivalent to samples prepared for the NPL, while the other half are fractionated. (b) Fractionation procedure for the PFL. Samples are separated by C18 flash chromatography and pooled to yield a total of eight fractions. Fractions are modestly concentrated twofold relative to crude extracts. (c) Workflow for pilot screening of the PFL versus the ESKAPE pathogens, pathogenic *Candida*, a hyperpermeable and efflux defective *E. coli*, and HEK293 cells. Edge effects were normalized by row–column normalization for fungal and bacterial screens, while a modified approach was employed for HEK cell screening (Supplementary Fig. S2) (d) Replica plot of a PFL screen versus *S. aureus*. The line graphs below and to the left of the scatterplot indicate whether the fitness effect observed in the scatterplot was associated with crude samples, hydrophilic (fr.1–4), or hydrophobic (fr.5–8) fractions. (e) Number of hits observed by assay and type of sample (crude or fraction). (f) Frequency of observation of bioactivity in one or more fractions for a given WAC extract by assay.

We developed a practical, cost-effective pipeline to generate microbial extracts (Fig. 2a) using low-cost materials to enable chemical screening. Understanding that growth media conditions can significantly impact the expression of specialized metabolites, we performed an extensive survey of conditions that in our hands offer broad culturability of soil bacteria, good compound production for many isolates, and a low background of UV-active material in LC-MS (Supplementary Fig. S3). One option is to prepare an extract library from isolates grown under many different conditions (Pan et al., 2019). For fungal strains and streptomycetes, solid media fermentation is often more successful (VanderMolen et al., 2013; Lajtai-Szabó et al., 2022). We found extensive media screening impractical for an academic lab and settled on the protocol described here as a compromise that works well in our hands. Microbes are grown on agar plates, and the cells and agar, impregnated with natural products, are extruded through 60 cc syringes (which can be washed and reused) to increase surface area and subsequently extracted with methanol. We have adapted a large can crusher (MasterCrush, https://www.carversoldeiron.com/) mounted on a simple wooden frame to facilitate agar extrusion. Methanol extraction is performed at room temperature with shaking over 16–24 h, and the solid material is removed by use of a milk filter. We explored alternative extraction solvents (butanol, acetone) in the context of known antibiotic producers and found that the solvents tested were similarly effective. Given that methanol lacks a reactive ketone as found in acetone; has a lower boiling point than butanol and is sufficiently volatile to minimize evaporation time; and is inexpensive, we chose to use methanol in our workflow. After drying in a centrifugal evaporator, the solid material is very dense and it dissolves slowly in DMSO over 2–3 days with periodic vortexing. While the long duration represents an opportunity for unstable compounds to degrade, such as the clipibicyclenes (Culp et al., 2022), we have had success identifying many classes of antibiotics with these approaches and view particular compound instability as a liability in our natural product discovery pipeline. Cleared extracts are aliquoted and form the basis of the NPL collection, which presently numbers ∼10 000 extracts. This collection has been a driving force of new chemical matter discovery in the laboratory (Cox et al., 2014; King et al., 2014; Perry et al., 2015; Gehrke et al., 2017; Yarlagadda, Medina, & Wright, 2020; Alder et al., 2022).

### Generation and Screening of a Pre-Fractionated Extract Library (PFL)

One of the drawbacks of the NPL is that it is often very viscous, which limits its use with modern acoustic-based dispensers used in HT screening labs. With this limitation in mind and given reports in the literature of greater hit rates observed by pre-fractionating whole cell extracts (Appleton et al., 2007; Wagenaar, 2008; Thornburg et al., 2018), we generated a pilot PFL from 381 isolates and 3 media-only controls.

Other published methods use resource intensive HPLC separation or solid phase extraction and collect many fractions (Appleton et al., 2007; Wagenaar, 2008; Thornburg et al., 2018). To optimize the process for an academic setting, we used medium pressure flash reverse phase chromatography and collected eight fractions across an acetonitrile gradient. NPL extracts (Fig. 2a), were separated by C18 flash chromatography using a water: acetonitrile gradient and formic acid ion pair in both mobile phases collecting eight fractions (Fig. 2a and b). Fraction 1 spans the elution range of DMSO, which contains the most hydrophilic compounds and those which co-elute with DMSO. Fraction 8 represents the most hydrophobic fractions, up to 100% of the acetonitrile mobile phase. The total number of fractions was chosen strategically to maintain a total of 10 samples per WAC strain in a 96-well screening plate (crude extract, CM, and eight fractions). Conditioned media was generated separately by growth of WAC strains in liquid culture with sterile filtration of the media. Columns 1 and 12 are left empty in our screening plates, allowing space in high-throughput assays for high–low controls. Initial screening plates included CM; however, due to a low–hit rate (Supplementary Fig. S4B), we discontinued CM preparation and these samples are omitted from subsequent analyses.

In a pilot assessment of the PFL, we screened fractions from 381 WAC isolates versus a panel of pathogens and other reporter organisms (*E. aerogenes, S. aureus, K. pneumoniae, A. baumannii, P. aeruginosa, E. faecium*; a clinical isolate of *E. coli*; two efflux-defective hyperpermeable *E. coli ΔtolCΔbamB*, one of which expresses the metallo-β-lactamase NDM1; and two pathogenic yeast species, *C. albicans*, and *C. auris*) for antimicrobial activity (Fig. 2c).

An example replicate data set is shown in Fig. 2d for *S. aureus*. Notably, growth inhibition activity is not equally distributed across fractions but is enriched in the more hydrophobic ones, indicating either similar bioactive compounds or a bias toward certain chemical properties in *S. aureus* antimicrobial natural products. This enrichment is most evident in *S.aureus*, but is observed with other organisms as well (Supplementary Fig. S4A).

Consistent with published studies (Appleton et al., 2007; Wagenaar, 2008; Thornburg et al., 2018), we observed an increase in the number of hits from fractions relative to crude extracts alone. However, this increase was modest (up to ∼ two fold) and organism-dependent (Fig. 2e; Supplementary Fig. S4C). Many hits appear in multiple fractions from a single WAC strain, consistent with either multiple bioactive compounds or individual compounds spanning multiple fractions (Fig. 2f).

**Fig. 3.**
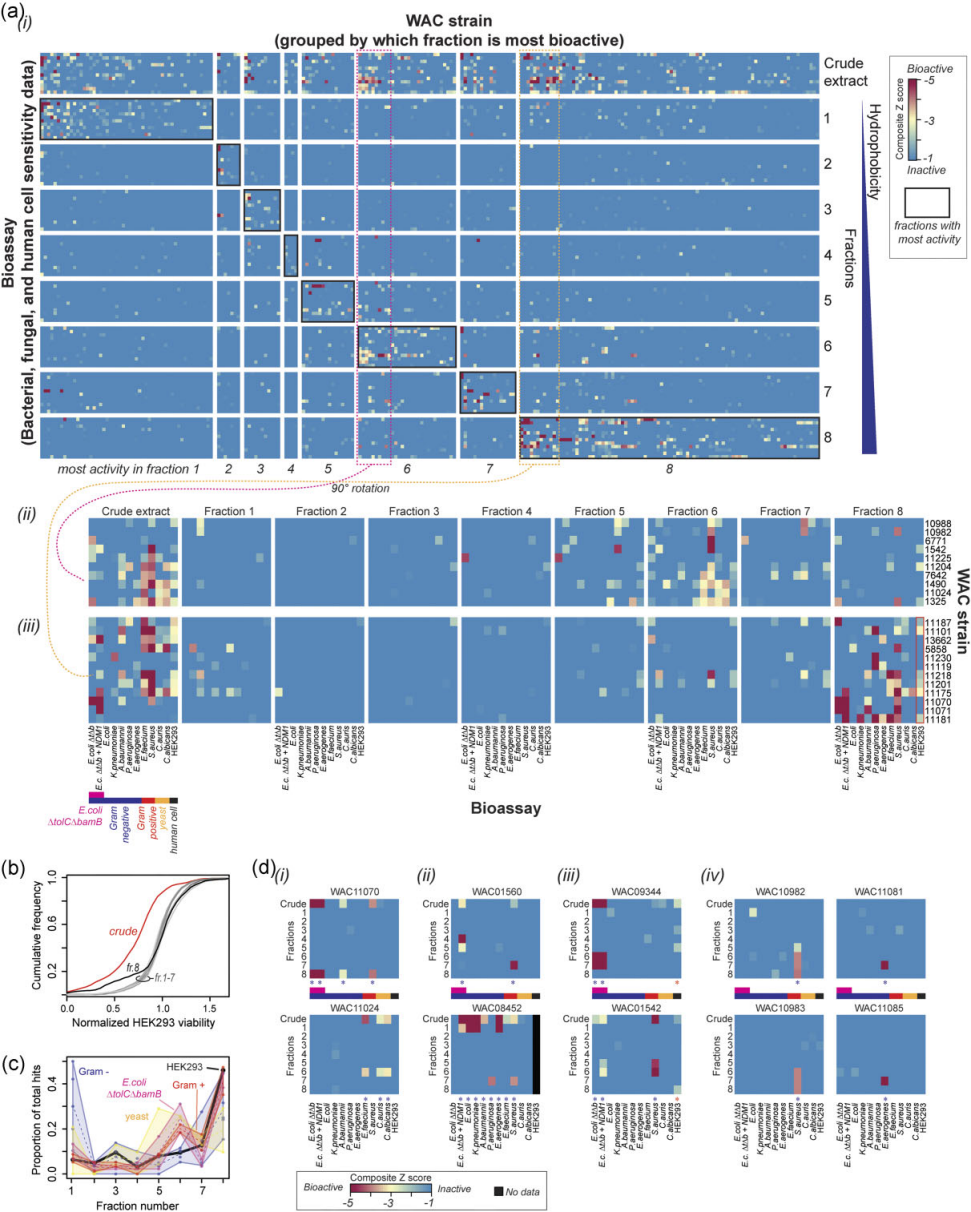
Prefractionation of whole cell extracts uncouples toxicity from antimicrobial activity and uncovers hidden activities. (a) Compilation of pilot screening data of PFL extracts and fractions for 381 WAC strains against a panel of sentinel organisms. (i) Overview of screening data (rows) versus WAC extracts and fractions (columns). Data are arranged by the type of chemical matter, whether crude extracts or particular fractions (*y*-axis). Each block along the *y*-axis includes data for all screens performed. Extracts are roughly arranged by the fraction within which the majority of bioactivity is observed (*x*-axis). Black boxes indicate where most activity in the fractions is observed. Data represent composite (average) *Z*-scores. Two regions of the data are indicated and shown in more detail. (ii) and (iii) Magnified view of the data indicated in panel (i), rotated 90°, to indicate the order in which the screening data is represented. Labels for screens are included for odd blocks of data for simplicity, but the order is consistent across fractions. (b) Cumulative frequency of toxic activity in HEK293 cells, as a function of chemical matter (crude extract or fractions). (c) Proportion of total hits (composite *Z* score ≤2) observed for various types of organisms, as a function of PFL fraction. Dashed lines represent the mean across organisms, while the shaded regions represent the high and low counts for individual screens/organisms within a given category. The solid black line with no accompanying range indicates average data from HEK293 cell screens. (d) Characteristic patterns of activity observed during prefractionation. (i) Spectrum of bioactivity in the crude extract is mirrored by activity in a single fraction. (ii) Pattern of bioactivity in crude extracts is split between multiple distinct fractions. (iii) Toxicity present in the crude extract is uncoupled from bioactivity in different fractions. (iv) Bioactivity absent in crude extracts is detected exclusively in fraction(s).

When we examined the entire dataset, consisting of 12 screens designed to capture antimicrobial, antifungal, and cytotoxic activities (Fig. 3a), we observed that bioactive hits were not equally distributed among the eight fractions. The most hydrophilic (fraction 1) and the most hydrophobic (fraction 8) fractions contain the most bioactivity (Supplementary Fig. S4B). Fraction 8 included the most toxic activity against HEK293 cells (Fig. 3b and c), albeit reduced compared to crude extract alone. Further, the active fraction distribution varies by target organism (Fig. 3c), with Gram-negative pathogens proportionally more affected by compounds in fraction 1, Gram-positive pathogens in fractions 6–7 (similar to efflux defective, hyperpermeable *E. coli*), and yeasts affected by compounds in fractions 5–6.

In some cases, the spectrum of activity observed in crude extracts is mirrored by that observed in a single fraction, suggesting a single or related class of molecules (WAC11070, WAC11024, Fig. 3d (i)). In other cases, the spectrum of activity in crude extracts represents multiple compounds, with the pattern of activity split between fractions (WAC01560, WAC08452, Fig. 3d (ii)). This observation is notable with “toxic” crude extracts that may otherwise have been lost when triaging screen data; in some cases, the antimicrobial compounds can be separated from the toxic compound (WAC09344, WAC01542, Fig. 3d (iii)). This “uncoupling” from toxicity is distinct from the general trend where crude extracts are more toxic than fractions, as this may simply be due to differing potency and compound dilution across fractions. In other cases, we note the appearance of activity in one or more fractions that were completely absent from crude material (WAC10982, WAC10983, WAC11081, WAC11085, Fig. 3d (iv)). Surprisingly, in some cases, this new-found activity is observed across multiple fractions, despite only a twofold enrichment relative to the crude material (WAC10982, WAC10983, Fig. 3d (iv)).

While the PFL is a relatively new resource in the laboratory, we already have evidence of success (Yarlagadda et al., 2021), with additional examples in the late stages of preparation.

### Prefractionation of Extracts Facilitates the Dereplication of Known Natural Products

The biggest obstacle to natural product discovery is the issue of rediscovery. Like others in the field of NP discovery, our pipeline involves a step to dereplicate or more accurately to “derisk” a given purification. Conclusive demonstration that a known compound is both present and responsible for observed bioactivity is time- and resource-limiting, and so we focus on rapid evaluation of risk of rediscovery. We have found the prefractionation of extracts to be particularly useful in the derisking process and here provide some examples of its application.

**Fig. 4.**
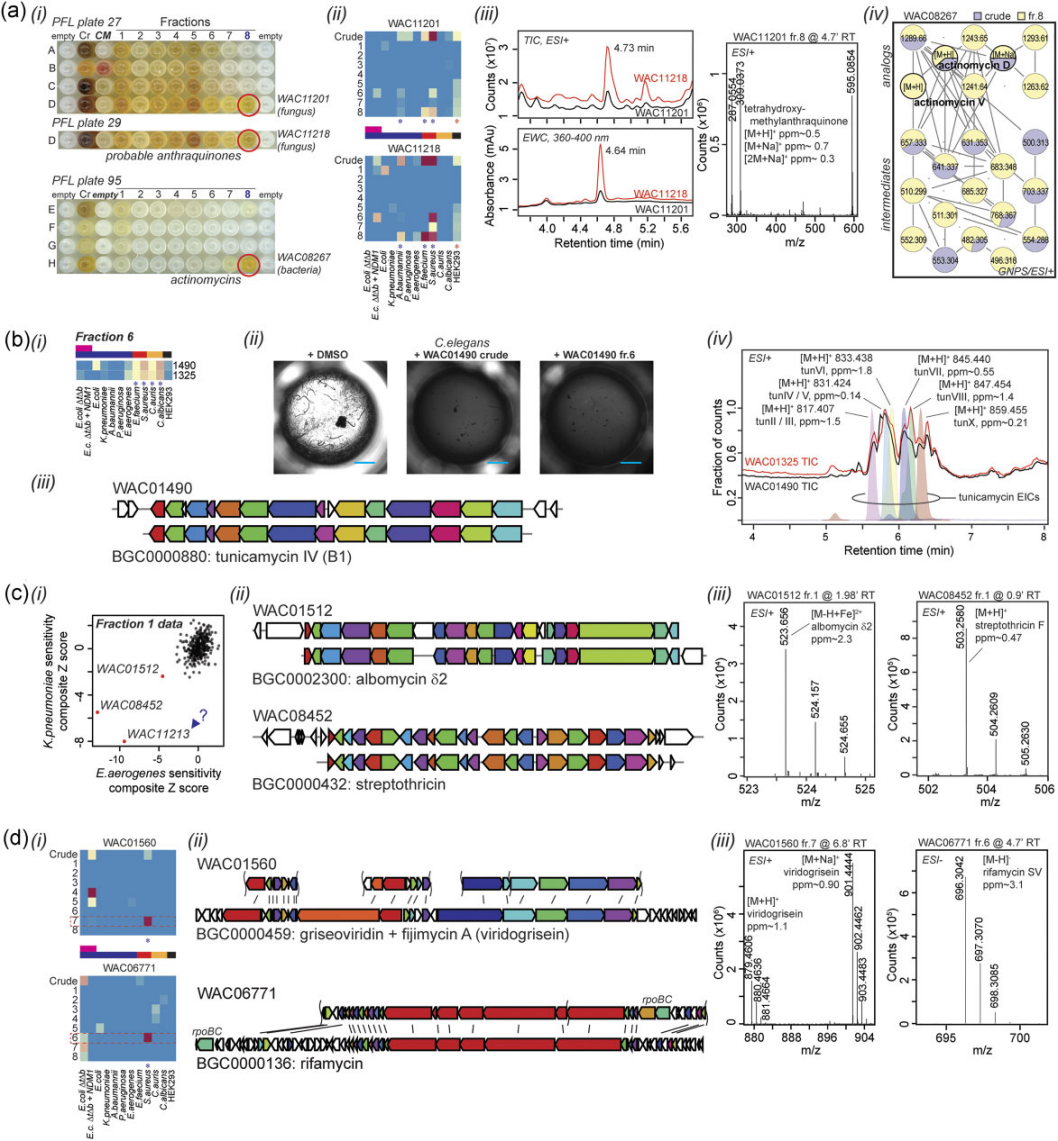
Prefractionation aids in the process of dereplication. (a) The pattern of color in a PFL plate can be suggestive of the presence of common natural products. (i) PFL plates with yellow material in fraction 8 from fungal (top) and bacterial (bottom) extracts often contain compounds consistent with anthraquinones and actinomycins, respectively. Conditioned media (CM) was initially included in PFL plates but ultimately discontinued due to low hit rate. Red circle, fractions of interest. Cr, crude. (ii) The fungal strains with similar patterns of color share a similar spectrum of activity. Color scale is the same as in Fig. 3. (iii) High resolution ESI-QTOF-MS data from fraction 8 of active fungal WAC strains. UV absorbance from 360–400 nm and a corresponding compound with a mass of 286.05 Da is suggestive of a tetrahydroxymethylanthraquinone. The UV detector and MS spectra are separated by a time interval of ∼ 0.1 min. (iv) Crude extracts and fraction 8 of WAC08267 contain actinomycins and intermediates. The network of related molecules was generated using the GNPS platform (Nothias et al., 2020). (b) Spectrum of activity of particular fractions can help dereplicate known compounds. (i and ii) Fraction 6 of two WAC strains is active against (i) Gram-positive bacteria, yeast; and (ii) *C. elegans*. The opacity of WAC01490 extract and fraction-containing wells, as compared to DMSO, is caused by “dietary” bacterial overgrowth in the absence of viable *C. elegans* to control the population. Scale bar, 1 mm. (iii) WAC01490 contains the BGC for tunicamycin (antiSMASH (Blin et al., 2023)). The tunicamycin IV cluster (MIBiG BGC000880 (Terlouw et al., 2023)) is shown for comparison. (iv) HR ESI-QTOF-MS data from fraction 6 of active WAC strains. Extracted ion chromatograms are shown in overlay for ions consistent with tunicamycins. (c) The fraction from which Gram-negative active hits are obtained can aid in dereplication of common natural products. (i) Scatterplot of composite *Z* scores for PFL fraction 1 samples versus two Gram-negative pathogens, *K. pneumoniae* and *E. aerogenes*. Bioactive outliers are indicated. A blue arrow represents a candidate for further purification with unknown identity. (ii) (Top) WAC01512 and (bottom) WAC08452 contain the BGCs for albomycin δ2 and streptothricin (antiSMASH (Blin et al., 2023)), respectively. The characterized MIBiG BGCs are shown for comparison (Terlouw et al., 2023). Curved lines indicate the boundaries of contigs. (iii) ESI-QTOF-MS of (left) WAC01512 and (right) WAC08452, indicating the presence of albomycin δ2 and streptothricin F, respectively. (d) PFL fractionation can improve detection of bioactive compounds. (i) Detection of bioactivity versus *S. aureus* is improved in fractions. (ii) (Top) WAC01560 and (bottom) WAC06771 contain the BGCs for griseoviridin/viridogrisein and rifamycin (antiSMASH (Blin et al., 2023)), respectively. The characterized MIBiG BGCs are shown for comparison (Terlouw et al., 2023). Curved lines indicate the boundaries of contigs. (iii) ESI-QTOF-MS of (left) WAC01560 and (right) WAC06771, indicating the presence of viridogrisein and rifamycin SV, respectively.

In some cases, visual inspection of a plate of extracts can provide clues as to common bioactive natural products (Fig. 4a). For example, extracts of fungal strains WAC11201 and WAC11218 contain a striking yellow color in fraction 8. Both extracts show a similar spectrum of activity. The strong UV signature in the 360–400 nm range is associated with mass signatures and fragmentation patterns (Supplementary Fig. S5A) consistent with anthraquinones, a common group of toxic molecules produced by yeasts (Masi & Evidente, 2020). Such evidence, while not conclusively establishing the compound identity, establish sufficient risk that we do not proceed with further purification with these samples. Similarly, bacterial extracts with intense yellow colors enriched in fraction 8 commonly contain actinomycins, as observed for WAC08267 (Fig. 4a; Supplementary Fig. S5B).

The spectrum of bioactivity observed in common fractions can also provide clues to aid in dereplication. WAC01490 was previously observed in the laboratory to produce tunicamycin, after discovery in a screen for anti-helminthics versus *C. elegans* (Fig. 4b). The WAC01490 genome contains the expected biosynthetic gene cluster (Blin et al., 2023) and tunicamycin can be observed by LC-MS (Fig. 4b, Supplementary Fig. S5C). Fraction 6 of WAC01490 has a similar spectrum of bioactivity to that in WAC01325 (Fig. 4b), suggesting it might also produce tunicamycin. Consistent with this hypothesis, WAC01325 has a similar LC-MS profile, and multiple tunicamycins can be observed in fraction 6.

In some cases, the fraction within which a given activity is observed can provide clues as to its identity. We have previously employed the Antibiotic Resistance Platform (Cox et al., 2017), an arrayed collection of resistance genes in an isogenic *E. coli* background, to identify the active components in many Gram-negative NPL hits. The most common natural products observed were albomycin, streptothricin, and streptomycin. All three compounds are hydrophilic and elute in fraction 1 of the PFL. Consistent with these results, our Gram-negative PFL screens are enriched for hits in fraction 1 (Fig. 3c). When we examine bioactivities of PFL fraction 1 samples versus *K. pneumoniae* and *E. aerogenes* (Fig. 4c), there are three outliers with potent bioactivity. Predictably, WAC01512, and WAC08452 contain the BGCs for albomycin and streptothricin, respectively. Correspondingly, ions consistent with albomycin δ2 and streptothricin F by exact mass and fragmentation pattern are observed in these fractions (Fig. 4c; Supplementary Fig. S5D and S5E). In contrast, WAC11213 is a fungal strain that does not make these common Gram-negative active compounds and is a candidate for further study (currently ongoing).

Fractionation offers the opportunity to purify and identify compounds that show weak or otherwise obscured activity in crude extracts. In WAC01560 and WAC06771, an anti-*S. aureus* activity was observed most strongly in fraction 6 or 7, respectively, with weak or absent activity in crude extracts (Fig. 4d). WAC01560 contains the BGC for viridogrisein, half of a described synergistic antibiotic pair (Asano & Adachi, 2006; Xie et al., 2012), while WAC06771 contains a BGC for a rifamycin, a well-established class of antibiotics. In both cases, ions corresponding with these compounds by exact mass and fragmentation were observed (Supplementary Fig. S6).

## Discussion

We describe here our experience building a collection of soil microbes and associated libraries of natural product extracts and a pre-fractionated library using simple, cost-effective, and easy to teach methodologies. The WAC and NPL have proved invaluable resources to the laboratory over the years, seeding multiple research projects (D'Costa et al., 2006; Perry et al., 2015; Robbins et al., 2016; Culp et al., 2020, 2022; Xu et al., 2020, 2022; Yarlagadda, Medina, Johnson, et al., 2020; Alder et al., 2022). In addition to their utility in discovery, these approaches have provided a valuable training opportunity for high school, undergraduate, and graduate students. Emphasizing the accessibility, other groups have employed similar public-based strategies to build up soil microbe collections. For example, the Tiny Earth initiative engages undergraduate students across multiple academic institutions in local soil microbe isolation, identification, and antimicrobial activity screening, all coupled to a central database (https://tinyearth.wisc.edu/) (Hurley et al., 2021)).

Our approach uses materials and techniques that are relatively inexpensive and accessible in most academic settings, democratizing the establishment of natural product libraries, which once were primarily the purview of the private sector.

Compound rediscovery is among the most challenging and costly disadvantages in a natural product discovery pipeline. In our experience, approximately 1 in 10 hits represent something new, while others represent rediscovered compounds. Numerous strategies have been employed to mitigate this issue—to establish the risk of rediscovery, with MS or MS2-based approaches, coupled with publically seeded compound databases (such as the Dictionary of Natural Products, https://dnp.chemnetbase.com/); and resistance-based platforms most used in our laboratory (Cox et al., 2017; Nothias et al., 2020). Where genome sequence is available, antiSMASH (Blin et al., 2023) has proved invaluable in establishing a short-list of antimicrobial candidates, though it is rarely cost- or time-effective to sequence a genome without prior derisking. Regardless, the dereplication problem remains a bottleneck and our approaches continue to evolve with the literature. For example, while we commonly correlate metabolomics data and bioactivity to narrow down candidate bioactive molecules on a case-by-case basis either manually or using software like Mass Profiler Professional (Agilent), resources like NP analyst represent more powerful and exciting additions to the community to integrate LC-MS and bioactivity data at scale (Lee et al., 2022).

In building a collection of natural product-producing microbes, reducing strain duplication is critical to lessen the frequency of compound rediscovery. We have had success with BOX-PCR and isolating organisms from diverse environmental samples to avoid the isolation of clones within samples. Prefractionation coupled with screening of sentinel organisms represents another tool to mitigate rediscovery, tying a spectrum of activity to additional chemical information on the hydrophobicity of a compound. As shown in the tunicamycin example, overlapping activity spectra observed between two or more strains/extracts may offer clues to the identity of an active compound and help dereplicate a collection.

The PFL has proved highly valuable despite the investment required. Aligning with the “One strain many compounds” concept (Bode et al., 2002), this work emphasizes the complexity of natural product extracts. Activity in a crude extract may represent multiple compounds. This complexity is of particular relevance when considering data from toxicity counterscreens. In our hands, much of the mammalian cell toxicity in natural product extracts is found in the most hydrophobic fractions. In contrast, many antimicrobial activities are more hydrophilic, suggesting toxicity in crude extracts is not always a red flag. Similarly, in the absence of fractionation information, one may assume the activity in a crude extract is linked to a single compound, while the reality may be that multiple compounds, each with a different spectrum of activity, may be present. Curiously, we observed some cases where the sum of activity in fractions appears much greater than that in crude extract. Whether this represents an antagonistic relationship between material in the more complex crude extracts, such as might be expected for an antimetabolite, or something more trivial remains to be seen.

Like many tools, dereplication with a pre-fractionated library is only as valuable as the source material. Rediscovery is still a problem but with a greater understanding of what compounds are commonly found where and what spectrum of activity they display across test organisms, its utility is increased. This observation is particularly true for fungal extracts, where historical knowledge is lacking, and represents a growing frontier for our laboratory and the community as a whole.

Our experience shows that establishing a collection of natural product-producing microbes and their extracts can identify novel chemical matter with a wide range of bioactivities. There is tremendous untapped potential available in microbial genomes that will enrich discoveries for decades to come. Establishing libraries of organisms that are difficult to culture, evolve in diverse environments, or that are found in the microbiomes of plants and animals, offer promising new areas of focus for collection. Networking these initiatives across the globe would provide a tremendous opportunity to explore biodiversity and identify the medicines of the future.

## Supplementary Material

kuad042_Supplemental_Files
