# Supplementary material for: Lessons from assembling a microbial natural product and pre-fractionated extract library in an academic laboratory
Source: J Ind Microbiol Biotechnol. 2023 Dec 5;50(1):kuad042. doi: 10.1093/jimb/kuad042 (PMC10724011; doi:10.1093/jimb/kuad042)
Supplement: kuad042_Supplemental_Files [file kuad042_supplemental_files.zip › Wright_revised supplemental data_20231211.pdf]

Supplementary data for "Lessons from assembling a microbial natural product and pre-fractionated extract library in an academic laboratory"

Michael A. Cook, Daniel Pallant, Linda Ejim, Arlene D. Sutherland, Xiaodong Wang, Jarrod W. Johnson, Susan McCusker, Xuefei Chen, Maya George, Sommer Chou, Kalinka Koteva, Wenliang Wang, Christian Hobson, Dirk Hackenberger, Nicholas Waglechner, Obi Ejim, Tracey Campbell, Ricardo Medina, Lesley T. MacNeil, Gerard D. Wright

Supplementary figure 1. Strain isolation conditions and de-duplication methods.

(A) Frequency of WAC strain isolation by the pre-treatment conditions described in Supplementary Tables 1-2. Data are represented on a log scale. Isolation conditions for various collections of donated strains are not documented here.

(B) Frequency of WAC strain isolation using the media described in Supplementary Table 3. Data are represented on a log scale. Isolation conditions for various collections of donated strains are not documented here.

(C) Example of the BOX-PCR strategy. Strains with similar colony morphology are compared based on the banding pattern observed by box PCR. Asterisks indicate strains with highly similar banding patterns. Only one isolate of such a group would be added to the strain collection.

(D) Example of fungal characterization by microscopy. Cells are visualized by lactophenol cotton blue staining. Common cellular structure, as observed for a suspected *Alternaria* sp., indicated possible strain duplication within a sample.

Supplementary figure 2. Layout of the PFL and normalization of PFL HEK293 cell screening data.

(A) Due to the layout of the PFL, with common chemical composition present within each column, data were normalized using trends across rows to estimate row and column edge effects. After modified row-"column" normalization, a remaining spatial effect was observed at the plate corners, which was normalized by distance from the plate corner. *Conditioned media was included in only a subset of plates and was excluded from the analysis.

(B)-(C) Box and whisker plots of the row trends and corner trends across the data set are shown for reference.

(D) Example corrective factors for each well are shown for illustration.

Supplementary figure 3. Optimization of conditions for WAC strain cultivation for extract generation.

(A) Overview of the workflow used to define media choice and cultivation conditions for NPL library generation. Ten strains known to produce antimicrobials were grown in parallel in liquid culture in five different media conditions for either 3 or 6 days at either 30 °C or 37 °C to assess growth and bioactive

secondary metabolite production. Filtered conditioned media (10% [v/v]) from these cultures was assayed for bioactivity versus 3 organisms: gram-negative *E. coli*, gram-positive *B.subtilis*, and yeast *S.cerevisiae*. For a subset of organisms, antimicrobial activity in liquid culture or solid agar was assessed versus the same panel of organisms. ONB, Oxoid nutrient broth (1 g Lab-Lemco powder, 2 g yeast extract, 5 g peptone, 5 g NaCl, 1L ddH2O, pH 7.4). MYM, maltose yeast extract media (4 g maltose, 4 g yeast extract, 10 g malt extract, 500 mL tap water, 500 mL ddH_2O) plus 2 mL R2 trace elements (40 mg zinc chloride, 200 mg iron chloride hexahydrate, 10 mg copper sulfate dihydrate, 10 mg manganese chloride tetrahydrate, 10 mg sodium tetraborate decahydrate, 10 mg ammonium heptamolybdate tetrahydrate, 1 L ddH_2O).

(B) Growth of 10 soil bacteria across media, temperature, and duration of time. Each point represents the average of two replicate cultures for a single organism. Growth was scored visually based on a relative turbity on a scale of 0 (no growth) to 3 (high turbity). Growth in SAM media at 30 °C for 6 days supported robust growth of all organisms (blue asterisk) and was chosen as the first step in the WAC cultivation workflow.

(C) Bioactivity of filtered conditioned media from cultures in (B) versus *E. coli*, *B. subtilis*, and *S. cerevisiae*. Data are shown for cultures grown at 30 °C for 6 days across 5 media types. One organism failed to grow in TSB and corresponding conditioned media was not tested (black). Conditioned media were assayed at 10% (v/v).

(D) Comparison of total wavelength chromatograms (190-600 nm) for SAM and Bennett's media. Bennett's media was chosen for secondary metabolite production due to a lower background signal in LC-MS.

(E) Bioactivity of conditioned media, extracted cell pellets, and extracted agar plates from 3 *Streptomyces* strains grown in Bennett's media at 30 °C for 6 days. Filtered conditioned media were assayed at 10% (v/v). Methanol extracts of cell pellet and solid media were dried and dissolved in DMSO and assayed at 2.5% (v/v).

Supplementary figure 4. Organism- and material-specific screening data.

(A) Replicate PFL screening data for all organisms. The line graphs below and to the left of the scatterplot indicate whether the fitness effect observed in the scatterplot was associated with crude samples, hydrophilic (fr.1-4), or hydrophobic (fr.5-8) fractions.

(B) Hit rate as a function of material assayed. Points represent hit rates (composite Z score < -2) for individual antimicrobial assays. Red lines indicate the mean hit rate.

(C) Frequency of identification of WAC strains as producing bioactive compounds in crude extracts or one or more fractions.

Supplementary figure 5. Fragmentation data for candidate compounds. (left) ESI-QTOF-MSMS data. Blue diamonds indicate the parent ion. Ions expected based on published spectra and expected molecular fragmentation are indicated in bold. (right) Molecular structure and expected or known fragmentation.

(A) Putative tetrahydroxymethylanthraquinone. Neutral losses are consistent with losses of CO or CH_3, as previously observed for this scaffold (Zhan et al., 2016). Substituents of the candidate structure with unknown positions on the heterocycle are shown adjacent to the scaffold.

(B) Actinomycin D. Sequential fragments of a substructure are indicated by color and dashed lines (Thomas et al., 1995)

(C) Tunicamycin VII. Fragments of the aglycone are indicated in gold text (right). Fragment masses shifted by mass differences consistent with changes in substructure are observed for the analogs in Figure 4B (not shown) (Tsvetanova et al., 2002).

(D) Albomycin δ2. Fragments of the 2+ ion, containing iron (Zakalyukina et al., 2022).

(E) Streptothricin F (Ji et al., 2008).

Supplementary figure 6. Fragmentation data for candidate compounds. (left) ESI-QTOF-MSMS data. Blue diamonds indicate the parent ion. Ions expected based on published spectra and expected molecular fragmentation are indicated in bold. (right) Molecular structure and expected or known fragmentation.

(A) Viridogrisein. Fragments expected from predicted peptide bond breakage are indicated (Krahn, 2019).

(B) Rifamycin SV. The diagnostic naphtalene core fragment is indicated (Koteva et al., 2018).

Supplementary table 1. Pre-treatments to enrich for bacterial spores. After pre-treatment, soil samples are diluted serially and cultured on agar plates of various compositions. Where possible, we have tried to include the primary reference or similar method. Any omissions or misattributions are unintentional. Pre-treatment conditions are reviewed in (Subramani & Aalbersberg, 2013).

Treatment	Method	Reference
Basic	Dry soil 5-7 days at room temp. in sterile petri dish<br>Suspend 1 g of soil in 10 mL sterile dH2O mixed by vortexing	(Williams & Cross, 1971)
Heat	Heat (dry) 1 g of soil at 110 °C for 1 h<br>Suspend in 10 mL of sterile dH_2O	(Nonomura & Ohara, 1969)
Heat and phenol	Heat (dry) 1 g of soil at 110 °C for 1 h<br>Add 4 mL of 1 % phenol<br>Shake mixture 200 rpm, 30 °C, 30 min<br>Add 5 mL of sterile dH_2O	Similar to (Hayakawa et al., 1991)
Phenol	Treat 1 g of soil with 1 % or 1.5 % phenol for 90 min	Similar to (Hayakawa et al., 1991)
Rehydration	Dry soil 5-7 days at room temp. in sterile petri dish<br>Mix 1 g of soil with 9 mL of 5 mM phosphate buffer, pH 7<br>Shake mixture 250 rpm, 37 °C, 90-120 min<br>Sonicate mixture 5 min, 40 kHz<br>Centrifuge mixture 15-20 min, 2600 rpm	Modified from (Hayakawa et al., 1991)
Benzethonium chloride (hyamine)	Add 29 mL of sterile dH_2O to 1 g soil<br>Shake mixture 180 rpm, 37 °C, 1 h<br>Add 0.01 % hyamine<br>Shake mixture 180 rpm, 30 °C, 30 min<br>Sonicate mixture 5 min, 40 kHz	Modified from (Hayakawa et al., 1991)
Media	Pre-incubation with HVA, EMB, SIM, or SEM media for 1-5 days at room temperature to 30 °C	Various

Supplementary table 2. Pre-treatments to enrich for fungal endophytes.

Treatment	Method	Reference
Basic	Air dry samples two weeks at room temperature<br>Wash and sonicate for 15 min to remove any soil residue<br>Sterilize surface sequentially:<br>- 5 % sodium hypochlorite, 7 min<br>- 2.5 % sodium thiosulfate, 10 min<br>- 75 % ethanol, 5 min<br>- wash in sterile dH_2O<br>- 10 % sodium bicarbonate, 10 min<br>Dry 100 °C, 10 min	(Arora et al., 2016; Qin et al., 2009)
Method 1: Sterile fragmentation	Aseptically fragment 2-3 g of surface sterilized sample<br>Place on selective media	(Qin et al., 2009)
Method 2: Calcium chloride paste	Mix 1 g of fragmented sample in a mortar with 0.5 g sterile powdered calcium carbonate<br>Place mixture in a petri dish<br>Flood with 4 mL of sterile dH_2O and mix to create a paste-like consistency<br>Incubate petri dish at 30 °C for 2 weeks<br>Air dry at room temperature<br>Place samples in a 50 mL tube with 50 mL of 10 mM phosphate buffer, pH 7<br>Incubate 1 h with periodic vortexing<br>Plate dilutions on selective media	(Qin et al., 2009)
Method 3:	Suspend 2-3 g of fragmented material in 25 mL of plant digestive enzyme solution (0.1 % macerozyme, 1 % cellulase, 0.7 M mannitol, 5 mM N-morpholinoethansulfonic acid, 9 mM calcium chloride, 65 mM dipotassium phosphate, pH 5.7, sterilized through 0.2 mM filter)<br>Shake at 250 rpm, 12 h, 30 °C<br>Clarify suspension by centrifugation, 200 xg, 10 min, 20 °C<br>Plate serial dilutions of supernatant on selective media	(Jiao et al., 2006)

Supplementary table 3. Media for enriching for and cultivating soil bacteria and fungal species. Antifungal compounds were included in some cases, including cycloheximide (20-50 mg/L), fluconazole (70 mg/L), amphotericin B (250 mg/L) to limit fungal contaminants or nalidixic acid (50 mg/L) to limit isolations of gram negative organisms. Other antibiotics were included to specifically enrich for resistant strains, such as gentamicin, linezolid, rifampicin, tetracycline, chloramphenicol, vancomycin, and bacitracin. Where possible, we have tried to include primary references. Any omissions or misattributions are unintentional.

Media	Composition	Reference
Humic acid vitamin agar (HVA), pH 5-12	1 g humic acid (in 10 mL 0.2N NaOH), 0.5 g disodium phosphate, 1.71 g potassium chloride, 0.05 g magnesium sulfate, 0.01 g iron(II) sulfate, 0.02 g calcium carbonate, B-vitamins*, 18 g agar. A range of pHs were surveyed, however, pH 6.5 or 7.2 are most commonly applied.	(Hayakawa & Nonomura, 1987)
Eosin methylene blue (EMB)	10 g peptone, 5 g lactose, 5 g sucrose, 2 g dipotassium phosphate, 13.5 g agar, 0.4 g eosin Y, 0.065 g methylene blue, pH 7.2	(Holt-Harris & Teague, 1916)
Enrichment broth	1 g arabinose, 1 g threonine, 5 g sodium chloride, 0.2 g magnesium sulfate, 1 g ammonium phosphate, 1 g dipotassium phosphate, 0.02 g yeast extract, 600 U/mL polymyxin B sulfate, 10 mg/mL gentamicin, 2 mg/mL vancomycin, 10 mg/mM cycloheximide	Modified from (Vanlaere et al., 2005)
Yeast extract agar (YEA)	5 g peptone, 3 g yeast extract, 15 g agar	Modified from (Lau et al., 2005)
Water-agar	15 g agar. Made up to 1 L with tap water.	(Lingappa & Lockwood, 1960)
Soil extract media (SEM)	1 L soil extract (1 kg soil in 50 mM NaOH, overnight at room temperature and filtered through cotton cloth; clarified at 18k rpm, and filtered at 0.2 mm), B-vitamins*, 15 g agar	(Hamaki et al., 2005)
Tap water yeast extract (TWYE)	0.5 g dipotassium phosphate, 0.25 g yeast extract, 18 g agar. Made up to 1 L with tap water. pH 7	(Crawford et al., 1993)
TWYE maple syrup	As TWYE recipe, with 8.3 mL of added maple syrup	This study
TWYE maple extract	As TWYE recipe, with plant extract instead of tap water (60 g finely chopped maple tree leaves and stems boiled in 1L dH_2O for 45 min and autoclaved)	(Qin et al., 2009)
Tryptic soy broth (TSB)	30 g tryptic soy broth, 15 g agar	We use a commercial source, BD Bacto (Fisher Scientific)
1/10 TSB	3 g tryptic soy broth, 15 g agar	Dilution of TSB
Streptomyces isolation media (SIM), pH 5-9	0.4 g casein, 1 g starch, 0.5 g potassium nitrate, 0.2 g dipotassium phosphate, 0.1 g magnesium sulfate, 0.1 g calcium carbonate, 15 g agar	(Kang et al., 1999)
Bennett's	10 g potato starch, 2 g casamino acids, 1.8 g yeast extract, 2 mL Czapek mineral mix**, 15 g agar, pH 6.8	(Jones, 1949)

ISP1	5 g casein peptone, 3 g yeast extract, 15 g agar	(Pridham & Gottlieb, 1948)
ISP4	10 g soluble starch, 1 g dipotassium phosphate, 1 g magnesium sulfate, 1 g sodium chloride, 2 g ammonium sulfate, 2 g calcium carbonate, 1 mg iron (II) sulfate, 1 mg manganese (II) chloride, 1 mg zinc sulfate, 15 g agar	(Kuester, 1959)
Maple syrup proline agar	5 mL maple syrup, 1 g proline, 1 g ammonium sulfate, 1 g sodium chloride, 2 g calcium chloride, 1 g dipotassium phosphate, 1 g magnesium sulfate, 15 g agar	Modified from (Qin et al., 2009)
Sodium Propionate-Asparagine-Plant Extract Agar (PAPE)	1 g sodium propionate, 0.2 g asparagine, 0.9 g potassium phosphate, 0.6 g dipotassium phosphate, 0.1 g magnesium sulfate heptahydrate, 0.2 g calcium chloride dihydrate, 1 mL plant extract (50 g Moroccan tea boiled in 500 mL water for 1 h, passed through milk filter, and evaporated under reduced pressure to yield 50 mL plant extract), 15 g agar, pH 7.2	(Li et al., 2011)
Cellulose proline agar	2.5 g cellulose, 2 g sodium pyruvate, 0.25 g potassium nitrate, 1 g proline, 0.2 g magnesium sulfate, 0.2 g dipotassium phosphate, 0.5 g calcium chloride, 1 mg iron (II) sulfate, 15 g agar	(Qin et al., 2009)
Chitin-cellulose	As for HVA, substituting chitin and cellulose for humic acid.	This study
Starch casein agar (SCA)	10 g soluble starch, 0.3 g casein, 2 g potassium nitrate, 0.05 g magnesium sulfate heptahydrate, 2 g dipotassium phosphate, 2 g sodium chloride, 0.02 g calcium carbonate, 0.01 g iron (II) sulfate heptahydrate, 18 g agar, pH 7.3	(Kuester & Williams, 1964)
Potato dextrose agar (PDA)	24 g potato dextrose broth, 15 g agar	We use a commercial source, Difco potato dextrose broth (Fisher Scientific)
*B-vitamins	0.5 mg thiamine-HCl, 0.5 mg riboflavin, 0.5 mg niacin, 0.5 mg pyridoxine-HCl, 0.5 mg inositol, 0.5 mg calcium-panthothenate, 0.5 mg p-aminobenzoic acid, 0.25 mg biotin	(Hayakawa & Nonomura, 1987)
**Czapek mineral mix	Per 100 mL, 10 g potassium chloride, 10 g magnesium sulfate heptahydrate, 12 g sodium nitrate, 0.4 g iron (II) sulfate heptahydrate, 200 mL HCl	(Jones, 1949)

Supplementary table 4. Oligonucleotide Primers

Primer	Sequence	Description
BOXA1R	5'- CTACGGCAAGGCGACGCTGACG	BOX-PCR primer
16S_BAC_F	5'-AGAGTTTGATCMTGGCTCAG	16S amplification primer
16S_BAC_R	5'-TACGGYTACCTTGTTACGACTT	16S amplification primer
18S_For	5' TTAGCATGGAATAATRRAATAGGA	18S amplification primer
18S_Rev	5' ATTGCAATGCYCTATCCCCA	18S amplification primer

Reference list

Arora, D., Sharma, N., Singamaneni, V., Sharma, V., Kushwaha, M., Abrol, V., Guru, S., Sharma, S., Gupta, A. P., Bhushan, S., Jaglan, S., & Gupta, P. (2016). Isolation and characterization of bioactive metabolites from Xylaria psidii, an endophytic fungus of the medicinal plant Aegle marmelos and their role in mitochondrial dependent apoptosis against pancreatic cancer cells. *Phytomedicine*, *23*(12), 1312-1320. https://doi.org/10.1016/j.phymed.2016.07.004

Crawford, D. L., Lynch, J. M., Whipps, J. M., & Ousley, M. A. (1993). Isolation and characterization of actinomycete antagonists of a fungal root pathogen. *Appl Environ Microbiol*, *59*(11), 3899-3905. https://doi.org/10.1128/aem.59.11.3899-3905.1993

Hamaki, T., Suzuki, M., Fudou, R., Jojima, Y., Kajiura, T., Tabuchi, A., Sen, K., & Shibai, H. (2005). Isolation of novel bacteria and actinomycetes using soil-extract agar medium. *J Biosci Bioeng*, *99*(5), 485-492. https://doi.org/10.1263/jbb.99.485

Hayakawa, M., Kajiura, T., & Nonomura, H. (1991). New methods for the highly selective isolation of Streptosporangium and Dactylosporangium from soil. *J. Ferment. Bioeng.*, *72*(5), 327-333.

Hayakawa, M., & Nonomura, H. (1987). Humic acid-vitamin agar, a new medium for the selective isolation of soil actinomycetes. *J. Ferment. Bioeng.*, *65*(5), 501-509.

Holt-Harris, J. E., & Teague, O. (1916). A New Culture Medium for the Isolation of Bacillus Typhosus from Stools. *J Infect Dis*, *18*(6), 596-600.

Ji, Z., Wei, S., Zhang, J., Wu, W., & Wang, M. (2008). Identification of streptothricin class antibiotics in the early-stage of antibiotics screening by electrospray ionization mass spectrometry. *J Antibiot (Tokyo)*, *61*(11), 660-667. https://doi.org/10.1038/ja.2008.93

Jiao, J. Y., Wang, H. X., Zeng, Y., & Shen, Y. M. (2006). Enrichment for microbes living in association with plant tissues. *J Appl Microbiol*, *100*(4), 830-837. https://doi.org/10.1111/j.1365-2672.2006.02830.x

Jones, K. L. (1949). Fresh Isolates of Actinomycetes in Which the Presence of Sporogenous Aerial Mycelia Is a Fluctuating Characteristic. *J Bacteriol*, *57*(2), 141-145. https://doi.org/10.1128/jb.57.2.141-145.1949

Kang, M., Kang, J., & Kim, E. (1999). Isolation and Characterization of Soil Streptomyces Involved in 2,4-Dichlorophenol Oxidation. *J Microbiol Biotech*, *9*(6), 877-880.

Koteva, K., Cox, G., Kelso, J. K., Surette, M. D., Zubyk, H. L., Ejim, L., Stogios, P., Savchenko, A., Sorensen, D., & Wright, G. D. (2018). Rox, a Rifamycin Resistance Enzyme with an Unprecedented Mechanism of Action. *Cell Chem Biol*, *25*(4), 403-412 e405. https://doi.org/10.1016/j.chembiol.2018.01.009

Krahn, S. (2019). *Etablierung eines industriellen Arbeitsablaufs zur Modulierung der Biosynthese von Sekundärmetaboliten in Actinobakterien* Justus Liebig University Giessen]. http://geb.uni-giessen.de/geb/volltexte/2019/14891/pdf/KrahnStefanie_2019_09_26.pdf

Kuester, E. (1959). Outline of a Comparative Study of Criteria Used in Characterization of the Actinomycetes. *Microbiology Society*, *9*(2).

Kuester, E., & Williams, S. T. (1964). Selection of Media for Isolation of Streptomycetes. *Nature*, *202*, 928-929. https://doi.org/10.1038/202928a0

Lau, S. C. K., Tsoi, M. M. Y., Li, X., Plakhotnikova, I., Dobretsov, S., Wong, P. K., Pawlik, J. R., & Qian, P. Y. (2005). Nonlabens tegetincola gen. nov., sp. nov., a novel member of the family Flavobacteriaceae isolated from a microbial mat in a subtropical estuary. *Int J Syst Evol Microbiol*, *55*(Pt 6), 2279-2283. https://doi.org/10.1099/ijs.0.63810-0

Li, J., Zhao, G. Z., Huang, H. Y., Zhu, W. Y., Lee, J. C., Xu, L. H., Kim, C. J., & Li, W. J. (2011). Nonomuraea endophytica sp. nov., an endophytic actinomycete isolated from Artemisia annua L. *Int J Syst Evol Microbiol*, *61*(Pt 4), 757-761. https://doi.org/10.1099/ijs.0.022558-0

Lingappa, Y., & Lockwood, J. L. (1960). Superior media for isolation of actinomycetes from soil. *Phytopathology*, *50*(9), 644.

Nonomura, H., & Ohara, Y. (1969). Distribution of actinomycetes in soil (VI). A culture method effective for both preferential isolation and enumeration of Microbispora and Streptosporangium strains in soil (part 1). *J. Ferment. Technol.*, *47*, 463-469.

Pridham, T. G., & Gottlieb, D. (1948). The Utilization of Carbon Compounds by Some Actinomycetales as an Aid for Species Determination. *J Bacteriol*, *56*(1), 107-114. https://doi.org/10.1128/jb.56.1.107-114.1948

Qin, S., Li, J., Chen, H. H., Zhao, G. Z., Zhu, W. Y., Jiang, C. L., Xu, L. H., & Li, W. J. (2009). Isolation, diversity, and antimicrobial activity of rare actinobacteria from medicinal plants of tropical rain forests in Xishuangbanna, China. *Appl Environ Microbiol*, *75*(19), 6176-6186. https://doi.org/10.1128/AEM.01034-09

Subramani, R., & Aalbersberg, W. (2013). Culturable rare Actinomycetes: diversity, isolation and marine natural product discovery. *Appl Microbiol Biotechnol*, *97*(21), 9291-9321. https://doi.org/10.1007/s00253-013-5229-7

Thomas, D., Morris, M., Curtis, J. M., & Boyd, R. K. (1995). Fragmentation Mechanisms of Protonated Actinomycins and Their Use in Structural Determination of Unknown Analogs. *Journal of Mass Spectrometry*, *30*(8), 1111-1125. https://doi.org/DOI 10.1002/jms.1190300808

Tsvetanova, B. C., Kiemle, D. J., & Price, N. P. (2002). Biosynthesis of tunicamycin and metabolic origin of the 11-carbon dialdose sugar, tunicamine. *J Biol Chem*, *277*(38), 35289-35296. https://doi.org/10.1074/jbc.M201345200

Vanlaere, E., Coenye, T., Samyn, E., Van den Plas, C., Govan, J., De Baets, F., De Boeck, K., Knoop, C., & Vandamme, P. (2005). A novel strategy for the isolation and identification of environmental Burkholderia cepacia complex bacteria. *FEMS Microbiol Lett*, *249*(2), 303-307. https://doi.org/10.1016/j.femsle.2005.06.026

Williams, S. T., & Cross, T. (1971). Actinomycetes. In C. Booth (Ed.), *Methods in Microbiology* (Vol. 4, pp. 295-334). Academic Press. https://doi.org/https://doi.org/10.1016/S0580-9517(09)70016-9

Zakalyukina, Y. V., Pavlov, N. A., Lukianov, D. A., Marina, V. I., Belozerova, O. A., Tashlitsky, V. N., Guglya, E. B., Osterman, I. A., & Biryukov, M. V. (2022). A New Albomycin-Producing Strain of Streptomyces globisporus subsp. globisporus May Provide Protection for Ants Messor structor. *Insects*, *13*(11). https://doi.org/10.3390/insects13111042

Zhan, C., Xiong, A., Shen, D., Yang, L., & Wang, Z. (2016). Characterization of the Principal Constituents of Danning Tablets, a Chinese Formula Consisting of Seven Herbs, by an UPLC-DAD-MS/MS Approach. *Molecules*, *21*(5). https://doi.org/10.3390/molecules21050631
