# Supplementary figures and images for: Lessons from assembling a microbial natural product and pre-fractionated extract library in an academic laboratory
Source: J Ind Microbiol Biotechnol. 2023 Dec 5;50(1):kuad042. doi: 10.1093/jimb/kuad042 (PMC10724011; doi:10.1093/jimb/kuad042)

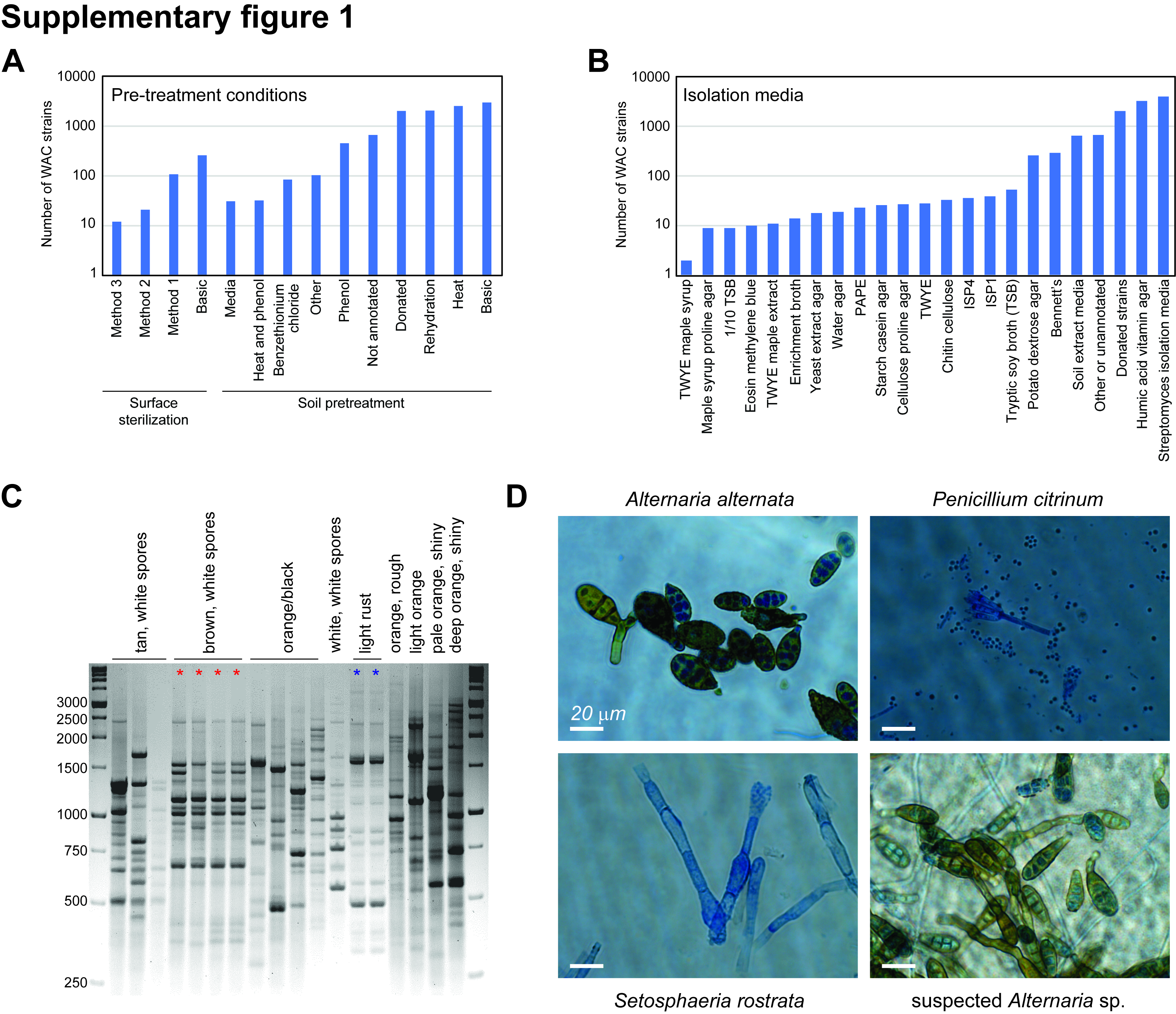

Supplement: kuad042_Supplemental_Files [file kuad042_supplemental_files.zip › Figure 1S - final.tif]

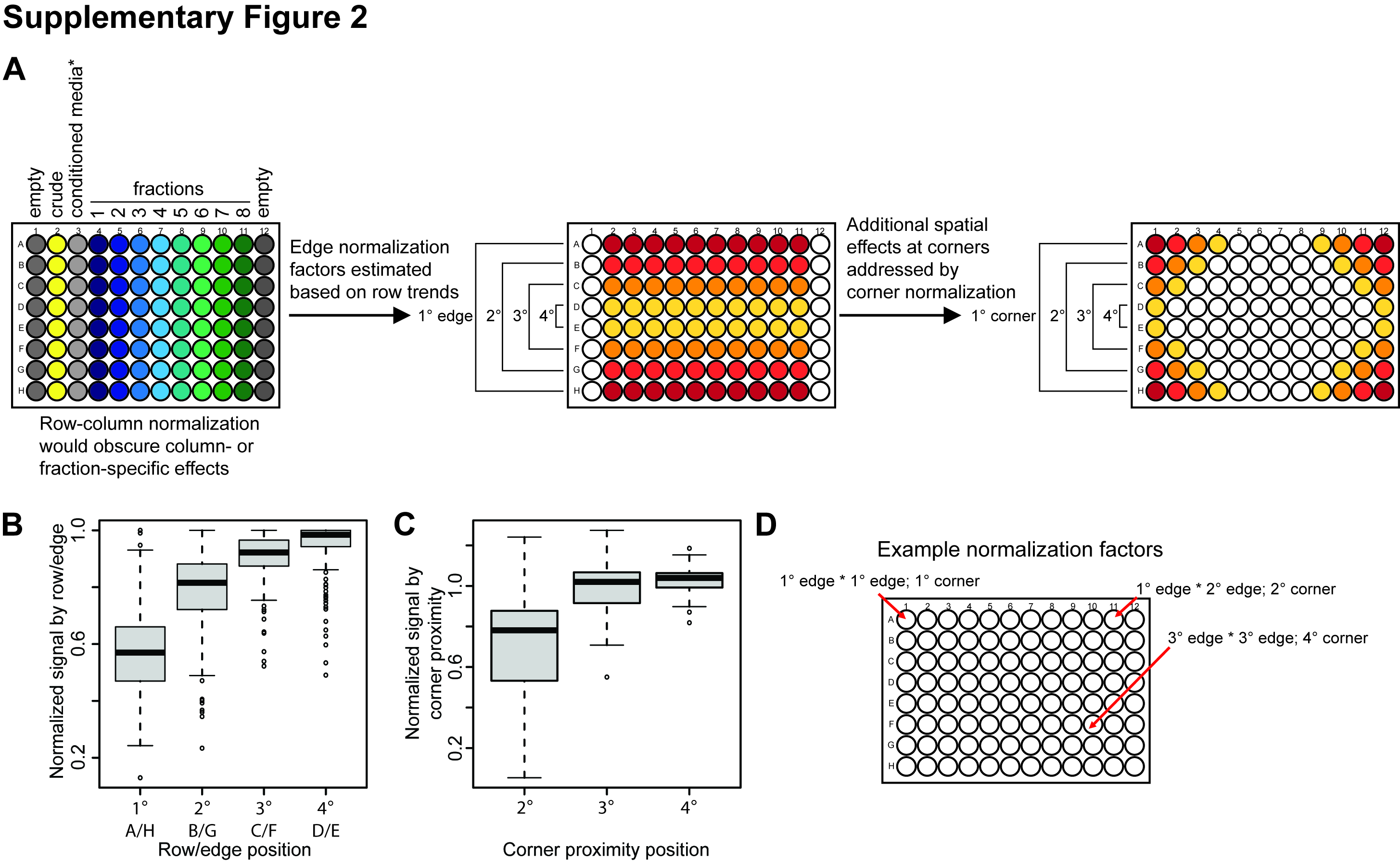

Supplement: kuad042_Supplemental_Files [file kuad042_supplemental_files.zip › figure 2S - final.tif]

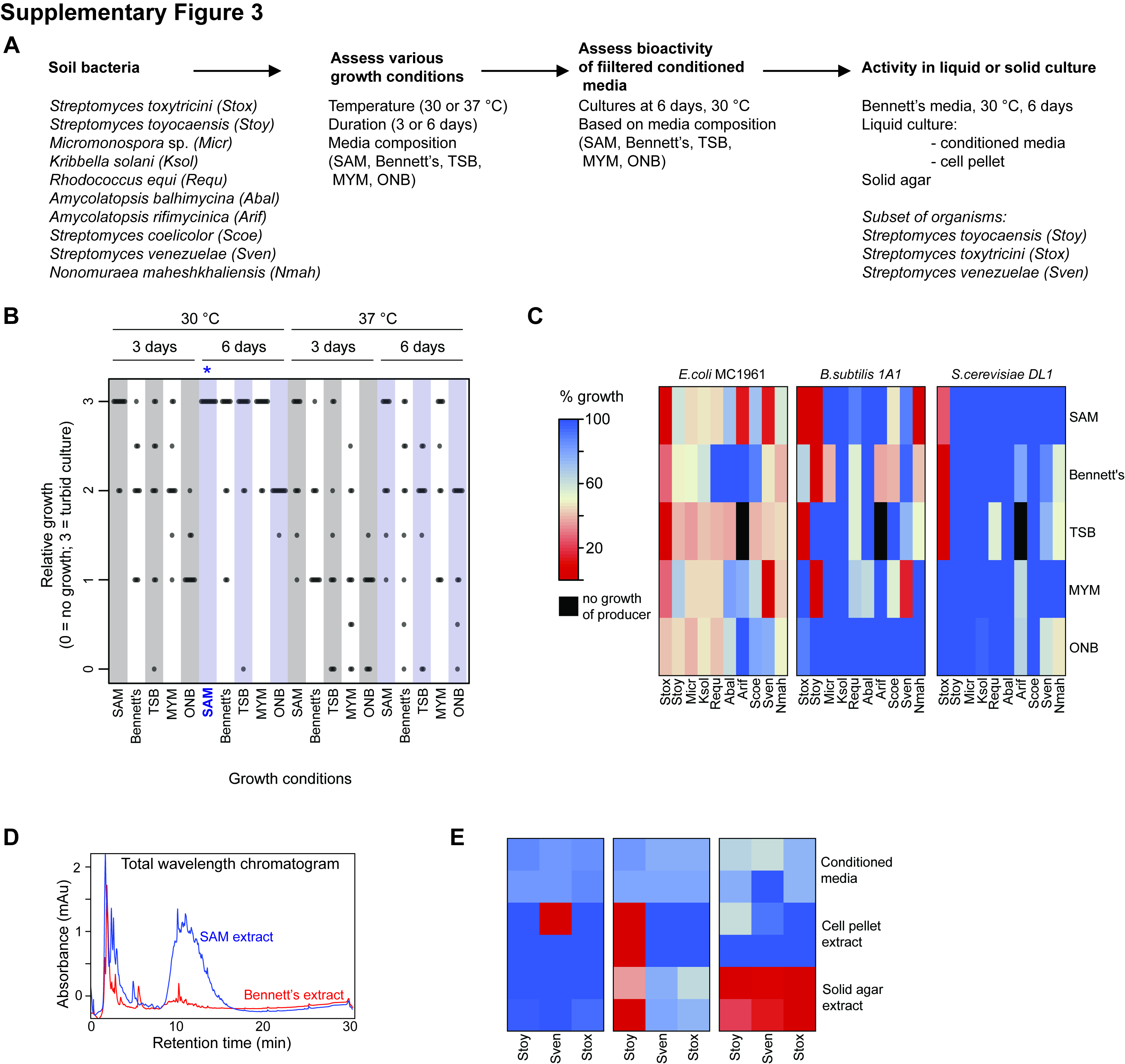

Supplement: kuad042_Supplemental_Files [file kuad042_supplemental_files.zip › Figure 3S - final.tif]

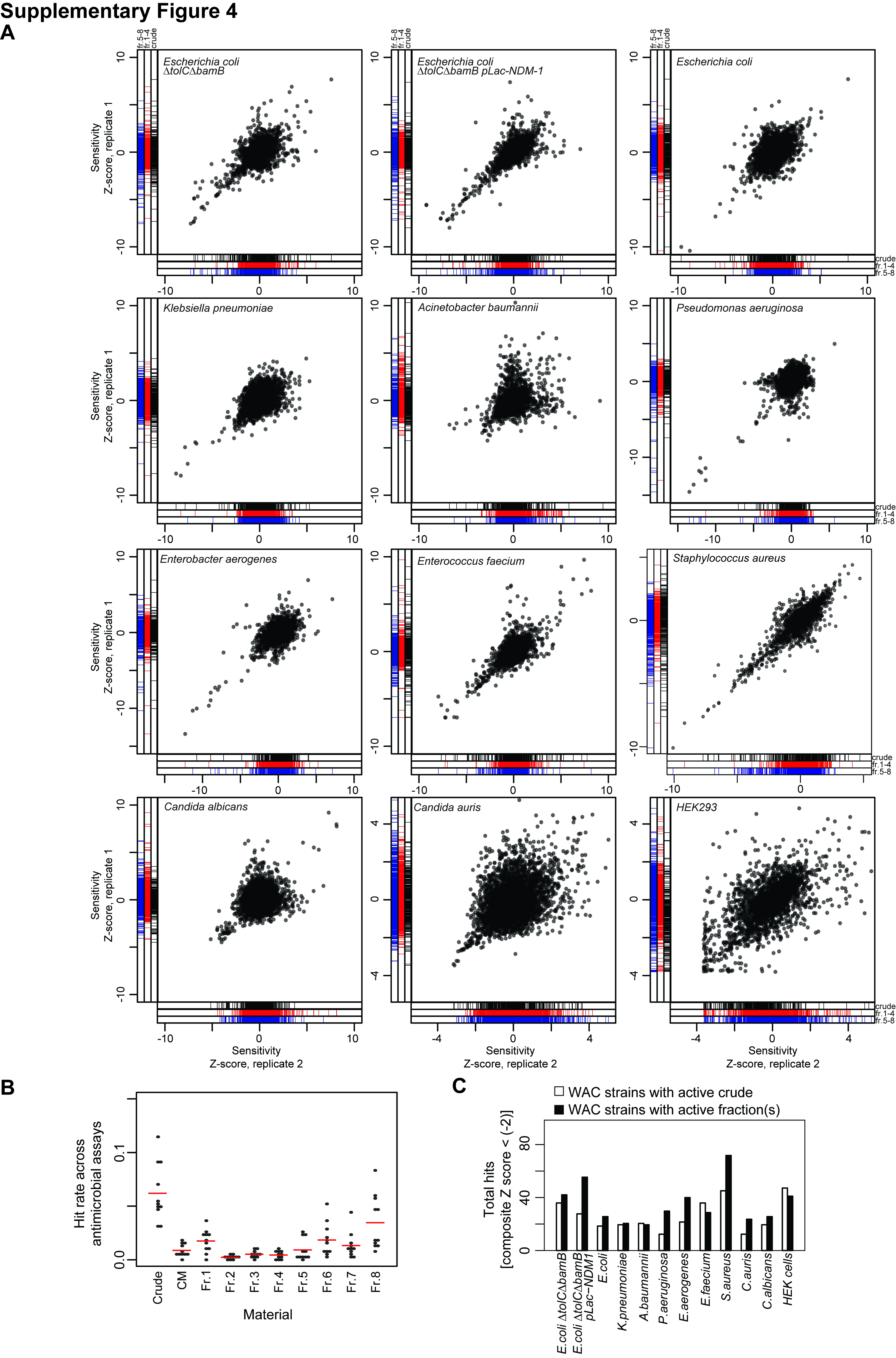

Supplement: kuad042_Supplemental_Files [file kuad042_supplemental_files.zip › Figure 4S - final.tif]

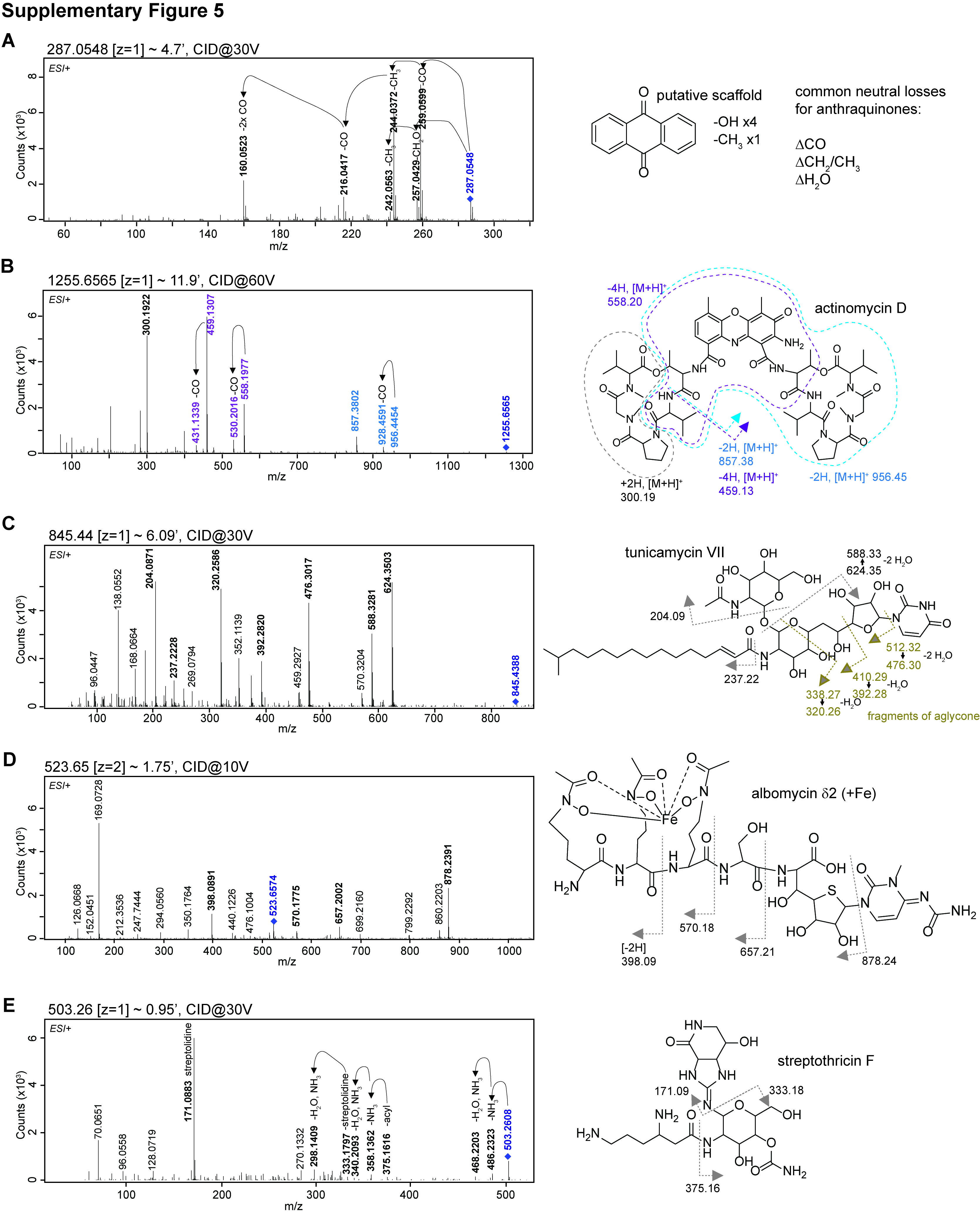

Supplement: kuad042_Supplemental_Files [file kuad042_supplemental_files.zip › figure 5S - final.tif]

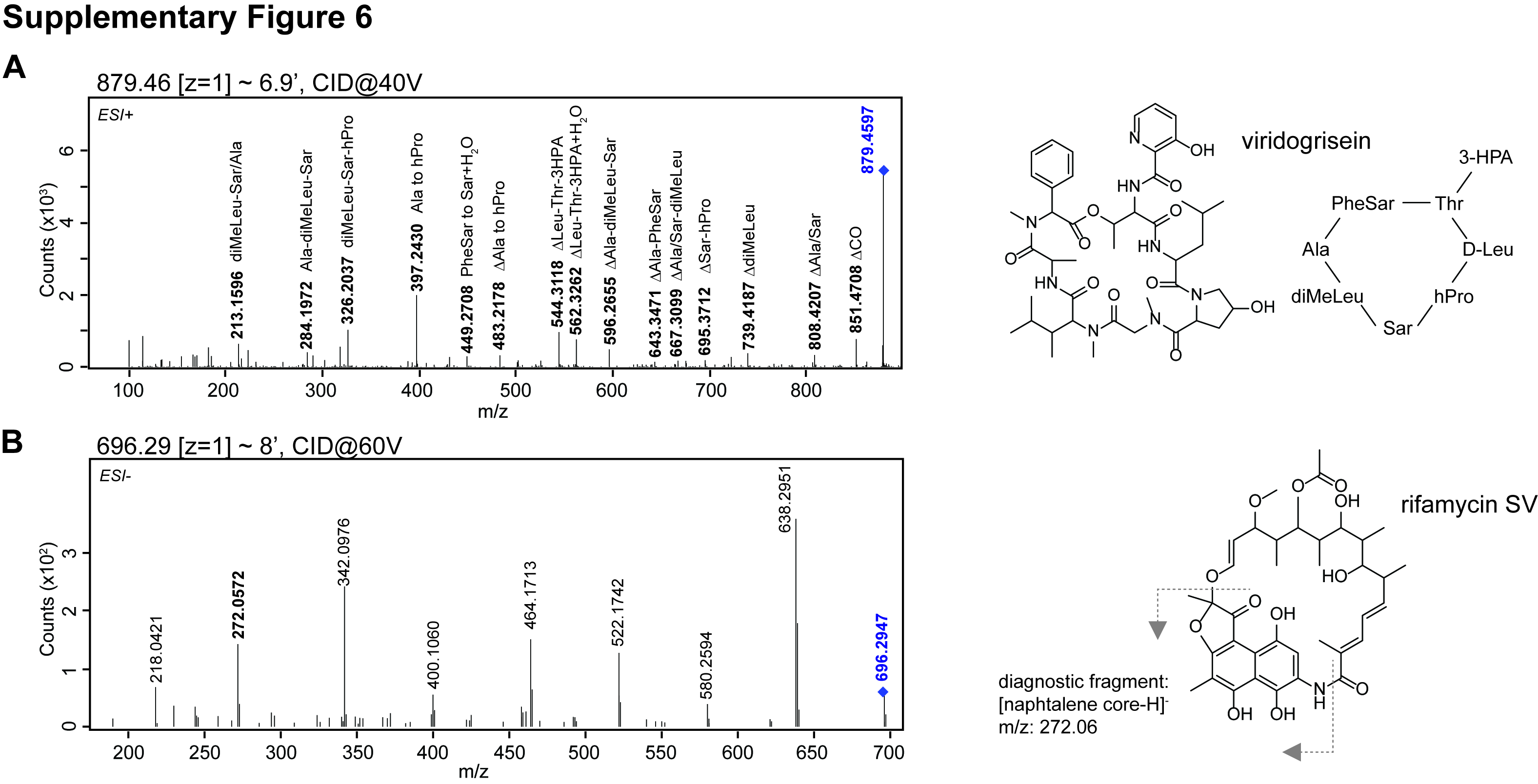

Supplement: kuad042_Supplemental_Files [file kuad042_supplemental_files.zip › figure 6S - final.tif]
